# Posterior Segment Ophthalmic Drug Delivery: Role of Muco-Adhesion with a Special Focus on Chitosan

**DOI:** 10.3390/pharmaceutics13101685

**Published:** 2021-10-14

**Authors:** Ayah Mohammad Burhan, Butsabarat Klahan, Wayne Cummins, Vanessa Andrés-Guerrero, Mark E. Byrne, Niall J. O’Reilly, Anuj Chauhan, Laurence Fitzhenry, Helen Hughes

**Affiliations:** 1 Ocular Therapeutics Research Group, Pharmaceutical and Molecular Biotechnology Research Centre, Waterford Institute of Technology, X91 K0EK Waterford, Ireland; wcummins@wit.ie (W.C.); NOREILLY@wit.ie (N.J.O.); LFITZHENRY@wit.ie (L.F.); hhughes@wit.ie (H.H.); 2 Innovation, Therapy and Pharmaceutical Development in Ophthalmology (InnOftal) Research Group, Department of Pharmaceutics and Food Technology, Faculty of Pharmacy, Sanitary Research Institute of the San Carlos Clinical Hospital (IdISSC), Complutense University of Madrid, 28040 Madrid, Spain; vandres@ucm.es; 3 Biomimetic & Biohybrid Materials, Biomedical Devices & Drug Delivery Laboratories, Department of Biomedical Engineering, Rowan University, Glassboro, NJ 08028, USA; byrnem@rowan.edu; 4 Chemical and Biological Engineering Department, Colorado School of Mines, Golden, CO 80401, USA; chauhan@mines.edu

**Keywords:** mucoadhesion, chitosan, chitosan coating for posterior eye segment drug delivery, posterior eye segment drug delivery, age macular degeneration, diabetic retinopathy, retinal drug delivery, permeation enhancement, topical drug delivery to the posterior eye segment, ophthalmic drug delivery, ocular drug delivery, chitosan coated drug delivery systems

## Abstract

Posterior segment eye diseases (PSEDs) including age macular degeneration (AMD) and diabetic retinopathy (DR) are amongst the major causes of irreversible blindness worldwide. Due to the numerous barriers encountered, highly invasive intravitreal (IVT) injections represent the primary route to deliver drugs to the posterior eye tissues. Thus, the potential of a more patient friendly topical route has been widely investigated. Mucoadhesive formulations can decrease precorneal clearance while prolonging precorneal residence. Thus, they are expected to enhance the chances of adherence to corneal and conjunctival surfaces and as such, enable increased delivery to the posterior eye segment. Among the mucoadhesive polymers available, chitosan is the most widely explored due to its outstanding mucoadhesive characteristics. In this review, the major PSEDs, their treatments, barriers to topical delivery, and routes of topical drug absorption to the posterior eye are presented. To enable the successful design of mucoadhesive ophthalmic drug delivery systems (DDSs), an overview of mucoadhesion, its theory, characterization, and considerations for ocular mucoadhesion is given. Furthermore, chitosan-based DDs that have been explored to promote topical drug delivery to the posterior eye segment are reviewed. Finally, challenges of successful preclinical to clinical translation of these DDSs for posterior eye drug delivery are discussed.

## 1. Introduction

The eye is classified into two main segments, the anterior segment (cornea-conjunctiva-iris-ciliary body-lens-aqueous humour), and the posterior segment (vitreous humour-sclera-choroid-retina-the optic nerve) [4,5]. Each of the two segments has its distinct anatomy and physiology, and many factors contribute to the hindrance of topical drug absorption to both segments. Despite the successful development of several promising therapeutic options such as growth factors, monoclonal antibodies, gene therapy, gene knockdown, and tissue engineering options, the delivery of these treatments to the ocular tissues remains highly challenging due to the highly protective and barrier anatomy and physiology of the eye tissues [6]. The eye is a pharmacokinetically isolated organ [7], and is very well protected by an efficient restrictive blood ocular barrier system against xenobiotics in blood. For this reason, the conventional oral and systemic routes of administration fail to deliver therapeutic concentrations of drugs to both the anterior as well as the posterior eye segments [6]. Consequently, the typical management of eye diseases involves the use of local ophthalmic drug delivery. Due to the larger number of barriers encountered, posterior segment drug absorption using topical administration remains more challenging. For this reason, IVT injections represent the main delivery route used in clinical practice for the treatment of posterior eye diseases such as AMD and DR among many others [8]. Despite their ability to achieve therapeutic drug concentrations in the posterior eye, IVT injections are highly invasive, and can cause serious complications including infection, cataracts, retinal detachment and vitreous haemorrhage [9]. In addition, the need for their frequent administration on a monthly basis further decreases patient compliance and leads to increased cost of treatment. Moreover, IVT injections have been reported to exacerbate the systemic side effects of anti-vascular endothelial growth factors (Anti VEGF) when used for their delivery to the posterior eye, causing life-threatening cardiovascular as well as cerebrovascular side effects including myocardial infarctions, transient ischemic attacks, deep vein thrombosis, pulmonary embolisms and thrombophlebitis [10].

**Figure 1:**
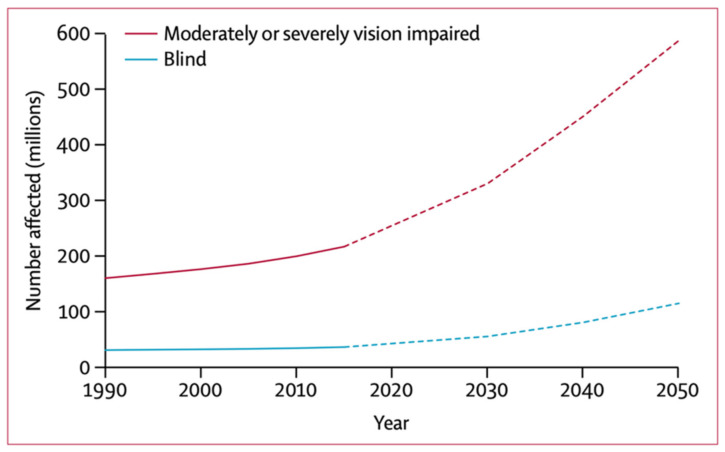
Global trends and predictions of numbers of people who are blind or moderately and severely vision impaired from 1990 to 2050. Reproduced from Bourne et al. [3], Elsevier, 2017.

Vision impairment can have substantial adverse effects on everyday activities of affected individuals affecting the mental wellbeing and quality of life of both patients as well as their families [1]. In addition, it has been implicated with higher risks of dementia as well as increased likelihood of accidents including falls and traffic crashes, i.e., higher mortality [1]. The increased demands for social care and costs of treatment also cause substantial economic impact for the affected communities. Latest 2020 prevalence data suggest that vision impairment associated annual global productivity loss in lower-middle income countries is estimated to be approximately USD 400 billion purchasing power parity [1]. Furthermore, the increase in the ageing population in a number of regions including Europe has led to a higher prevalence of age-related causes of blindness [1]. For example, around 67 million in the EU are currently affected by AMD which represents the main cause of visual impairment and blindness in Europe where the numbers are expected to increase by 15% by 2050 [2]. Thus, vision impairment is a global public priority that highly influences both developed as well as developing communities, and maintenance of proper vision is a global health priority [1]. The plot in Figure 1, measured in millions of people affected, illustrates the estimated number of people worldwide that have moderate or severe vision impairment and blindness, from 1990 to 2050 [3].

Thus, there is an unmet clinical need for the development of less invasive and more patient friendly delivery alternatives. Due to its high market potential and less invasive nature, topical drug delivery to the posterior eye segment is an active research area. Several approaches including nanotechnology drug delivery systems, mucoadhesion, and permeation-enhancement based techniques have been investigated to potentiate topical drug absorption to the posterior eye segment tissues [11,12,13,14]. In this review, mucoadhesive drug delivery approaches that aim to enhance topical ophthalmic drug delivery to the posterior eye segment will be reviewed.

Mucoadhesion in ocular delivery utilizes the ability of mucoadhesive polymers to bind and interact with the mucosal layer of the tear film. It has been estimated that out of the total drug amount that overcomes precorneal clearance, about 5–10% can enter the eye depending on the drug’s permeability coefficient and molecular weight [15]. Thus, owing to the ability of mucoadhesion to prevent rapid precorneal clearance and increase precorneal residence time, it is currently being explored for its potential in increasing chances of corneal and conjunctival absorption to the posterior segment, i.e., promoting non-invasive drug delivery to the posterior eye segment following topical administration.

Among the numerous available polymers, chitosan (CS) has been the most widely explored for potentiating topical drug delivery to the posterior eye segment. CS is a positively charged biocompatible and biodegradable polymer that is obtained from chitin (the second most abundant polymer in nature) using partial alkaline deacetylation yielding *N*-acetyl-d glucosamine units linked to d-glucosamine units via 1,4-glycosidic linkages [16,17]. Chitosan’s cationic charge allows its exploitation in many drug delivery applications, and is responsible for its potential in gene delivery, vaccine adjuvant properties, antimicrobial activities, as well as formation of ionic interactions with a vast number of negatively charged polymers [16,17,18,19]. Furthermore, electrostatic interactions between chitosan’s positive charges and the negatively charged sialic acid residues of mucus account for its mucoadhesive potential, which has been widely investigated to increase residence time, and achieve sustained drug release profiles [18]. In addition to facilitating mucoadhesion, CS also acts as a permeation enhancer [19]. This activity is again attributed to its positive charge, which mediates its interactions with the involved cellular membranes leading to the opening of intercellular tight junctions and a reduction in the transepithelial electrical resistance (TER), and an increase in paracellular permeability, thus creating permeation pathways for drugs to traverse these mucosal cells [16,17].

In this review, the major posterior eye diseases, their main treatments, the potential benefits of exploring topical ophthalmic drug delivery, and the barriers that need to be overcome for enabling topical drug delivery to the posterior eye segment will be presented. Furthermore, the currently reported pathways that could be harnessed for posterior eye delivery using the topical route are discussed. For the successful design of mucoadhesive ophthalmic DDSs, an overview of mucoadhesion, including fundamentals, characterization techniques and mucoadhesion considerations for ocular delivery are presented. Furthermore, CS-based mucosal delivery approaches that have been explored to promote topical drug delivery to the posterior eye segment are reviewed. Finally, the factors and challenges that need to be considered for successful preclinical to clinical translation of Cs based mucoadhesive DDSs for posterior eye drug delivery will be presented.

## 2. Posterior Segment Eye Diseases: A Global Health Crisis

Among the various PSEDs, DR and AMD are the most common. The estimated number of people living with diabetes worldwide in 2017 was 451 million, and this figure is expected to reach 693 million by 2045 [20]. Another major posterior eye disease that causes irreversible blindness in elderly populations is AMD. AMD is one of the most sight threatening PSEDs. It is reported to be the main causative agent of central visual loss and irreversible blindness in industrialized countries in individuals above 55 years old [21,22,23]. Systemic review indicated that 8.7% of the worldwide population has AMD. Moreover, this number reached 196 million in 2020 and is projected to extend to 288 million in 2040 [24]. Hence, these trends demonstrate that there are an increasing number of people who will present with diseases in the posterior part of the eye, if prompt action is not taken.

### 2.1. Posterior Segment Eye Diseases

**Figure 2:**
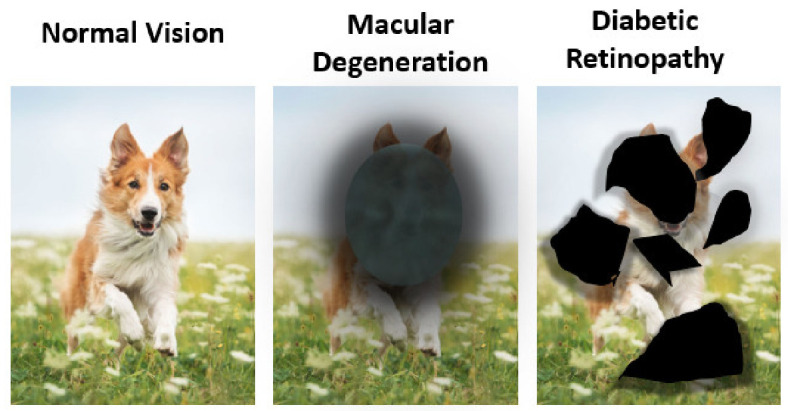
An image comparing normal vision, vision in AMD, and vision in DR.

The retina is one of the most important tissues of the posterior eye segment whose principle function is the conversion of light into neural signals [25], and most posterior segment eye diseases including AMD and DR involve retinal abnormalities that interfere with the normal visual function as illustrated in Figure 2 [26], and as will be discussed in Section 2.1.1 and Section 2.1.2.

#### 2.1.1. Age Related Macular Degeneration (AMD)

**Figure 3:**
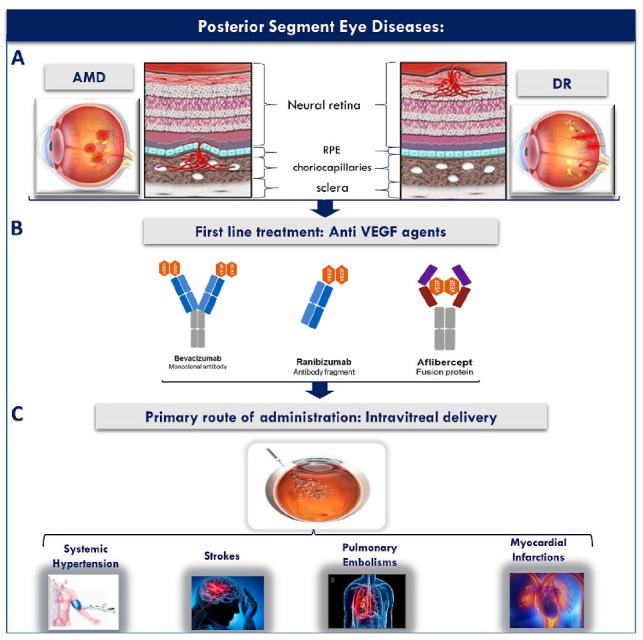
(**A**) Hallmark differences between angiogenesis in AMD and DR. In AMD neovascularization occurs in the sub retina or from the choriocapillaris, whereas in DR, neovascularisations occur at the retinal surface, (**B**) Showing the structure of Anti VEGF agents which represent the first line treatment of neovascular AMD and DR, (**C**) Showing the effect of intravitreal delivery on exacerbating the systemic side effects of Anti VEGF drugs.

AMD is a neurodegenerative disorder involving the macula in which patients suffer from progressive deterioration in their visual function [22,27]. The macula accounts for 4% of the retinal area, and is the only retinal location where 20/20 vision is possible. It is responsible for visual acuity and discrimination. It has the highest density of retinal ganglion cells (around 30%), and most of its photoreceptor cells which mediate transduction of light into nerve impulses are of the cone type, which is responsible for colour vision [28,29]. The photoreceptors within the macula have a very high metabolic activity with the highest oxygen consumption throughout the body. This high oxygen consumption, in addition to the excessive light irradiation exposure of the macula, is associated with the production of oxygen free radicals. With aging, the ability of the macula to maintain protection against different stresses, including free radicals, deteriorates, which accounts for the degenerative changes observed in AMD [22,29]. AMD has two main types (1) Dry AMD, also called atrophic AMD or geographic atrophy, and (2) Wet AMD, also called neovascular or exudative AMD. Wet AMD or neovascular AMD is characterized by choroidal neovascularization (CNV) which is a type of angiogenesis associated with the choroid as illustrated in Figure 3A. In CNV, the growth of new abnormal blood vessels (choriocapillaries) from the choroid usually results in Bruch’s membrane penetration leading to scarring, RPE detachment, exudations, and haemorrhage [21,30,31,32].

#### 2.1.2. Diabetic Retinopathy (DR)

DR is an important eye disease that can lead to loss of vision. The longer the patient had diabetes, the more likely they can develop DR, since DR is caused by high blood sugar levels. People who live with DR notice symptoms such as seeing an increasing number of floaters and seeing black or dark areas in their field of vision (Figure 2). Currently, DR is a significant cause of vision impairment. Globally, 2.6 million people were living with DR in 2015 and this number has reached 3.2 million in 2020 [33]. Moreover, the projection of people in the world with DR tends to increase continuously in the near future if it is not treated properly [34]. To overcome this problem, innovative drug development strategies are essential for protecting against sight loss in people.

### 2.2. Conventional Therapies and Their Drawbacks

In terms of DR and wet AMD treatment, there are several treatment strategies including laser photocoagulation, vitreoretinal surgery, and IVT administration of anti-VEGF drugs as well as steroids [35,36].

#### 2.2.1. Anti-VEGF Agents

There is a wide variety of anti-VEGF agents that have been proven effective for the management of wet AMD and DR as summarized in Table 1.

The development and use of anti-VEGF medications such as bevacizumab, ranibizumab and aflibercept have generated an impressive amount of research since this drugs can stop the growth of the abnormal vessels generated from the vascular endothelial growth factor (VEGF) encountered in wet AMD or DR. Bevacizumab, a monoclonal antibody, was investigated first as a systemic intravenous injection for cancer treatment and then as an IVT injection for wet AMD [37]. A study focusing on IVT injections of bevacizumab to 79 patients who have subfoveal neovascular AMD illustrated that no significant ocular or systemic side effects were observed at 1 month (1.25 mg dose of bevacizumab), and more than 55% of those patients had a reduction in baseline retinal thickness after 1 week of injection. In terms of aflibercept, a number of scholars have pointed out that a 2 mg dose of aflibercept via IVT injections monthly or every 2 months displayed similar efficacy and safety outcomes as a 0.5 mg dose monthly of ranibizumab [41]. Moreover, most of clinical research on ranibizumab suggests the effectiveness of IVT ranibizumab for treating eye disease is similar to bevacizumab and aflibercept. In other words, ranibizumab 0.5 mg IVT injection enhances visual acuity (34%) of people who have neovascular AMD (*n* = 176) [39].

Nowadays, numerous scholars have turned their attention to find out new and longer lasting drugs for treating patients with wet AMD instead of bevacizumab, aflibercept and ranibizumab, above described, which need to be injected in the eye 4–8 weeks. Hence, recent studies have explored the use of brolucizumab and conbercept, the novel FDA approved anti-VEGF agents, for the treatment of AMD and DR as these drugs could help reduction in eye damage associated with a number of injections (Table 1). To be specific, brolucizumab is a humanized single-chain antibody fragment which is the smallest functional unit of an antibody. This allows its delivery in a greater molar dose compared to large molecules. Additionally, it can prolong the duration of action due to more effective tissue penetration of the small molecule drug [46,51,52]. In addition, brolucizumab offers both greater fluid resolution, a longer duration of therapeutic action, vs. aflibercept, and the ability to maintain eligible wet AMD patients on a three-month dosing interval immediately after a three-month loading phase, leading to patient care improvement in wet AMD [51]. Laboratory-based study on the efficacy of brolucizumab in patients (*n =* 1817) who had choroidal neovascularization has shown that brolucizumab was not inferior to aflibercept in mean best-corrected visual acuity (BCVA) change from baseline to week 48. In addition, more than 50% of brolucizumab-treated eyes received an injection every 12 weeks with 6 mg were maintained through week 48. Additionally, greater central subfield thickness reductions were observed from brolucizumab 6 mg (least squares (LS) mean—193.8 µm) as compared to aflibercept (LS mean—143.9 µm) [44]. In another study, the work of Pravin et al., focusing on comparing the efficacy of brolucizumab with aflibercept to treat neovascular AMD demonstrated a greater proportion of brolucizumab-treated eyes (61%, 6 mg/50 µL) achieved simultaneous resolution retinal fluid (IRF and SRF) at weeks 40 compared to aflibercept (35%, 2 mg/50 µL), indicating a better fluid control and resolution of brolucizumab in comparison to aflibercept in the retina [51]. Furthermore, a number of studies on conbercept (Table 1) have also shown promise for the drug’s ability to treat neovascular AMD since conbercept has higher binding affinity of a ligand to VEGF-A (Kd = 0.5 pM) than ranibizumab and bevacizumab, resulting in greater bimolecular interactions between the target molecule and the ligand [49,53]. Besides, the IVT half-life of conbercept in vitreous (4.2 days) is longer than ranibizumab (2.9 days), which can lead to more effective inhibitory effect against VEGF [54,55]. Gao et al. found that IVT injection of conbercept 0.5 mg reduced the central retinal thickness (CRT) at months 9 to 12 in the patients with exudative AMD (*n =* 106) [56]. Similarly, Bai et al. studied the safety of intravitreal conbercept in patients with retinopathy of prematurity (ROP) (*n =* 24) [57]. Their study revealed that 40 out of 48 eyes (83.3%) had a regression of disease at an average of 3.5 weeks after receiving intravitreal conbercept (IVC) only once (10 mg/mL) with no signs of lens opacity, vitreous haemorrhage or retinal detachment, which indicates an effective IVC for ROP [57]. Therefore, these results, as mentioned previously, indicate the efficacy of both brolucizumab and conbercept as novel VEGF inhibitors for the treatment of posterior segment eye diseases, especially AMD and DR, due to their longer duration of action and reduced need for injections.

The last few years have seen an increased interested in developing devices for ocular drug delivery to address posterior segment eye diseases, including ocular implants [58,59,60]. As an example of the port delivery system (PDS) of non-biodegradable implants for AMD treatment, consider the study of Peter et al., which could prolong release of anti-VEGF agents [58]. In their study, the port delivery system with ranibizumab is designed to be implanted in sclera of AMD patients (*n* = 232), and sustain drug release in the vitreous. The data provide convincing evidence showing that 100 mg/mL of the PDS of ranibizumab had a similar outcome of visual acuity as monthly intravitreal ranibizumab 0.5-mg injections [58]. In addition, the PDS strategy could extend the drug release that the median time to first implant refill for the PDS 100 mg/mL was up to 15 months, indicating a reduction in total number of ranibizumab injections. Moreover, previous studies have demonstrated the development of nano-formulation for sustained delivery of anti-VEGF by embedment in an ocular implant. Badiee et al., in their study of bevacizumab-loaded chitosan nanoparticles inserted in a matrix of hyaluronic acid and zinc sulfate for CNV treatment, found that 79 nm sized chitosan nanoparticles with 67% of entrapment efficiency illustrated long-term sustained release of the drug from the carrier over two months, which can introduce as a promising carrier in the treatment of PSEDs [61]. Taken altogether, a number of scholars have shown that intravitreal injection is the most attractive method for ocular implants. Nevertheless, this method can potentially cause several side effects, such as endopthalmitis and vitreous hemorrhage, when repeated injections are given [62]. Additionally, owing to pars plana stab incision before implant insertion, this makes patients to the risk of complications and serious infections inside the eyes, especially retinal detachment, during implantation [58,63].

#### 2.2.2. Corticosteroids

Numerous researchers have investigated the ability of corticosteroids to treat several ocular conditions affecting the posterior eye segment due to their anti-inflammatory as well as anti-neovascularization properties. Indeed, corticosteroids have the ability to inhibit both VEGF expression and the activation of matrix metalloproteinase which play an important role in the angiogenesis processes, resulting in downregulation of inflammatory agents [64]. Although IVT corticosteroids have been extensively studied in the management of posterior eye diseases owing to overcoming the blood-ocular barrier and other barriers related to topical administration, corticosteroid-induced intraocular pressure elevation and cataract formation are major ocular side effects that patients encounter when using corticosteroids either topically or systemically [65,66,67]. Additionally, other severe drawbacks related to IVT corticosteroid injections include detachment of the retina, vitreous hemorrhage and infectious endopthalmitis [68,69].

There are several widely used ophthalmic corticosteroids for treatment of posterior segment eye diseases such as dexamethasone (DEX), triamcinolone and fluocinolone acetonide. DEX has become a key aspect of the most potent corticosteroid agents. Over the years, a number of studies have reported that three combinations, which were dexamethasone, verteporfin photodynamic therapy and anti-VEGF agents, presented a good outcome in treating CNV lesions from AMD, leading to minimizing a number of anti-VEGF injections [70,71]. The prospective, noncomparative and interventional case studies of 104 patients with CNV owing to AMD were evaluated [71]. The patients (*n* = 140) were firstly received verteporfin PDT, followed by injection of intravitreal DEX (800 mg) and bevacizumab (1.5 mg). Noticeable and sustained enhancements were observed after one triple therapy cycle. Eighteen of those patients required an additional IVT of bevacizumab for retinal modelling. In contrast, five patients received a second triple treatment owing to remaining CNV activity. In addition, mean visual acuity improved in most patients, from 20/126 to 20/85, and mean retinal thickness reduced from 463.5 at baseline to 281 µm at follow-up after 40 weeks of the treatment. Besides, in terms of triamcinolone acetonide (TA), previous research has supported that due to the large particle size of TA, it has a longer duration of action in the vitreous compared to DEX [72,73]. For this reason, several studies have explored intravitreal TA for treatment of AMD. For example, the clinical study involved 30 eyes of AMD patients with occult or minimally classic CNV was investigated [74]. The patients were treated with only photodynamic therapy (PDT) or combined intravitreal TA (12 mg) plus PDT treatment. The results demonstrated a stable mean visual acuity in the PDT plus intravitreal TA. Whilst, there was a significant decline in visual acuity in the PDT alone group. Moreover, the number of treatments in the PDA plus intravitreal TA was two-fold lower than the PDT group (1.13), supporting a reduction in a number of treatment frequency at 12 months with combination between PDT and intravitreal TA.

In conclusion, as discussed above, IVT injections represent the standard administration route that is employed clinically for the delivery of medications to the posterior eye tissues [75,76]. Additionally, the IVT anti-VEGF injections are currently the most effective treatment used for wet AMD since the IVT route not only maintains high concentrations of drug in the vitreous humor through repeated injections given every month or two, but also delivers the drug directly to the site of action in the posterior segment of the eye [36,75,77]. Nonetheless, IVT injections are highly invasive and present several risks such as infections, cataracts, retinal toxicity from injected agents, retinal detachment, vascular occlusion, intraocular inflammation, and vitreous haemorrhage resulting in inconvenience to patients [9,78]. Furthermore, the IVT route has been reported to exacerbate the systemic side effects of anti-VEGF agents leading to life threatening systemic cardiovascular as well as cerebrovascular side effects including myocardial infarctions, transient ischemic attacks, deep vein thrombosis, pulmonary embolisms and thrombophlebitis as illustrated in Figure 3C [10]. Hence, there is a clear unmet medical need to investigate safer, less invasive and more patient friendly approaches that can lead to improved patient compliance and therapeutic efficacy.

## 3. Topical Drug Delivery to the Posterior Eye Segment

The typical management of anterior as well as posterior eye diseases involves the use of local ophthalmic drug delivery. The eye offers multiple potential entry routes through which ocular drugs may be delivered. Posterior segment delivery can be achieved in several ways, including topically. Indeed, topical administration refers to the application of medication to the surface of the tear film of the eye, and this route is widely used for drug delivery to treat eye diseases in the anterior segment. Nonetheless, it remains a major challenge to deliver drugs topically for treating posterior segment diseases such as AMD and DR [79]. Therefore, a considerable amount of research has focused on using this mode of drug delivery to deliver drugs to the back of the eye. Efforts in ocular drug delivery are focused on prolonging the contact of drug delivery systems with the ocular surface, sustaining the release of drugs and enhancing corneal permeability. For these reasons, various ocular drug delivery strategies namely mucoadhesives, nanoparticles and soft contact lenses, have been investigated, and several methods of ocular drug administration to posterior segments of the eye have been developed [80,81,82]. Among the various ocular drug delivery routes, topical administration might be the best approach to overcoming several drawbacks, since drugs can be administered non invasively to the surface of the eye [83]. This overcomes substantial issues issues with drug administration via injections or implants, which are invasive approaches, such as desegmentation of the implant, accidental injection into the crystalline lens and migration of the implant into the anterior chamber [84]. In addition, most available topical ocular preparations are in the form of aqueous ophthalmic formulations [85]. Although topically applied drugs as commercial eye drops are commonly used by patients due to their ease of usage, low interference with vision and non-invasiveness, overusing eye drops in the long term can put your eye health at risk, leading to several drawbacks namely eye redness, irritation and dry eye. In some cases, this can cause glaucoma due to eye drops containing steroid, with consequent vision loss and blindness [86,87,88,89]. Thus, careful design of non-irritant and non-toxic topical ophthalmic preparations that do not interfere with the long-term eye physiology is crucial.

In this section, a brief account of the eye’s anatomy and the main challenges that face topical drug absorption to the posterior eye will be given. After this, the recently explored pathways for topical drug absorption into the posterior eye segment will be presented.

### 3.1. Eye Anatomy and Hurdles of Topical Drug Delivery to the Posterior Eye Segment

**Figure 4:**
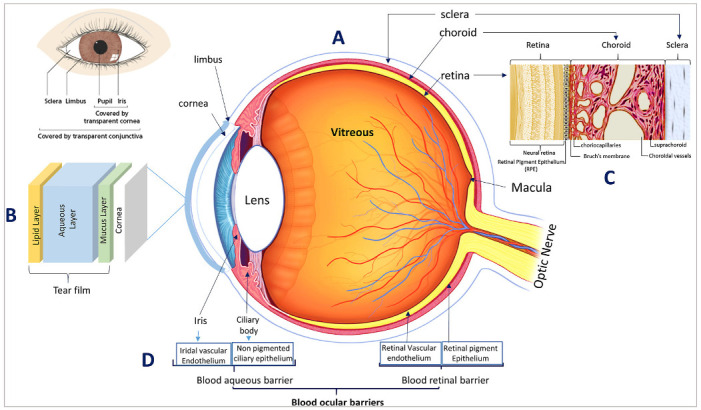
Anatomy of the eye showing (**A**) Anterior and posterior eye segments, (**B**) Tear film composition, (**C**) Posterior segment detailed tissues, and (**D**) Structural composition of blood ocular barriers.

Anatomically, the eye can be divided into two main segments, the anterior segment and the posterior segment, each of which has distinct anatomy and physiology as illustrated in Figure 4 [4,5].

The anterior eye segment consists of the cornea, the conjunctiva, the iris, the ciliary body, the lens, and the aqueous humour, and the posterior eye segment consists of the vitreous humour, sclera, choroid, retina, and the optic nerve [8]. An excellent overview of the various eye tissues, their anatomy, and physiology is described elsewhere [6,90,91]. In this section, the main barriers hindering topical drug absorption to the posterior eye will be presented.

The major PSEDs including AMD and DR are associated with abnormalities in the retinal tissues, and thus their management requires efficient retinal drug delivery. In comparison to the anterior eye segment, drug delivery to the posterior segment using topical administration is much more challenging. When an ophthalmic DDS is topically applied to the ocular surface, it encounters two main types of barriers (1) physiological barriers, and (2) anatomical barriers [91].

Physiological barriers including blinking, nasolacrimal drainage, and tear turn over lead to the rapid removal of topically applied drugs from the ocular surface. This results in a substantially reduced drug dosage on the ocular surface whose absorption is further hindered by the various anatomical eye barriers [6].

One of the first barriers that topically applied ocular drug delivery systems encounters is the external tear film layer. An excellent overview of the ocular tear film, its structure, composition and dynamics has been presented by Mark D.P. Willcox et al. [92]. The tear film is composed of an outermost lipid layer (0.1 μm), interior to which lies the aqueous layer (7 μm), after which comes the innermost mucus layer (3–30 μm) as illustrated Figure 4B [25]. Mucus aids in the adhesion of the tear film to the eye surface, thus enabling it to perform its crucial defensive, nutritional, mechanical, as well as optical functions [25]. Due to its defensive role, the tear film is thought to act as a barrier against ocular drug absorption and a limiting factor against drug diffusion [91]. However, the mucus component of the tear film is currently explored as a target for mucoadhesive drug delivery systems to serve as non-invasive delivery alternatives to deliver drugs topically to the anterior and posterior eye segments, as will be discussed in Section 4, Section 5 and Section 6.

Permeation of topically applied drugsthrough the corneal and conjunctival epithelia representsthe main rate limiting step for drug absorption to the posterior eye segment [15], and depends on three important factors which are (1) the drug’s concentration gradient, (2) the drug’s lipophilic/hydrophilic character, and (3) molecular weight. On considering the first factor, the rapid physiological washout of topically applied drugs, as well as the limited ocular surface area, lead to decreased concentration gradients across the ocular surface, hence decreasing chances of drug permeation across the corneal and conjunctival epithelia [15].

**Figure 5:**
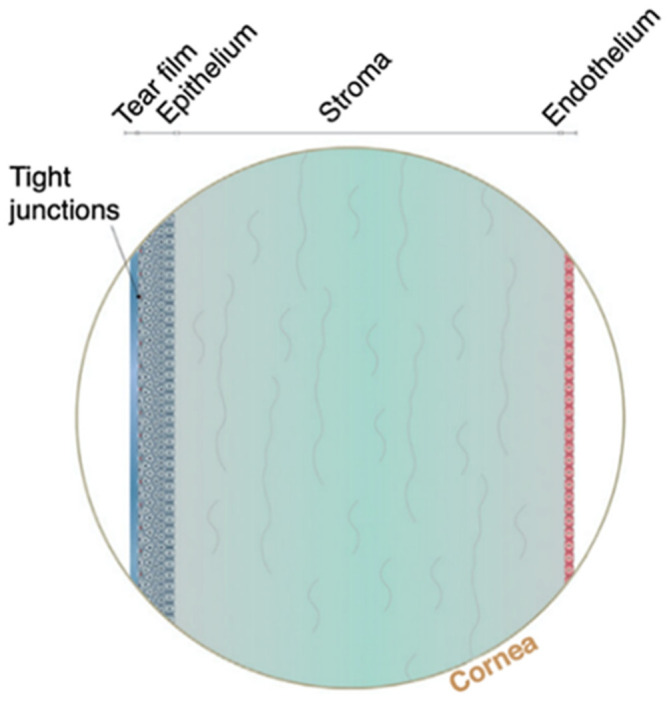
Cross section of the corneal tissue barrier for drug penetration after topical installation. Reproduced from Subrizi al. [76], Elsevier, 2019.

With respect to the corneal tissue, it represents a main barrier of the topical route, as shown in Figure 5. The cornea consists of three main layers-epithelium, stroma and endothelium-with each layer presenting different challenges for drug permeation (Figure 5) [79]. The lipophilic nature of the corneal epithelium, presents a large barrier for permeation of hydrophilic molecules, whereas the hydrophilic corneal stroma acts as a permeability barrier to lipophilic drug molecules. The innermost layer of the cornea is the corneal endothelium, which is a monolayer of hexagonal endothelial cells that adjust water influx into the cornea and a barrier between the cornea and aqueous humour [93,94].

In comparison to the corneal epithelia, the conjunctival epithelia are 16 fold higher in pore density. In addition, they have relatively higher intercellular pore diameters (3.0 nm ± 1.6) that can allow the passage of up to 5–10 KDa sized molecules [15]. On the other hand, the corneal intercellular pore diameter is estimated to be only 2.0 nm ± 0.2, and can allow only molecules of less than 500 Dalton to pass through. For this reason, the conjunctival epithelia has 15–25% higher permeability and is, therefore, a promising pathway that has been implicated with the absorption of hydrophilic drugs. Due to these favourable reasons, the conjunctival penetration pathway is receiving attention for its potential as an alternative to the corneal route, and for its potential in promoting topical drug absorption to the posterior eye segment, as will be discussed in Section 3.2 [15,90]. Furthermore, their leaky epithelial cells that can permeate large molecular weight and hydrophilic molecules are interesting to explore for the absorption of proteins and peptides [6]. However, it is important to note that a portion of the molecules transporting through the conjunctiva can be cleared via blood and lymphatic clearance into systemic circulation reducing the net flux across the conjunctiva into the deeper ocular tissue. Considering drug related properties, it is reported that hydrophobic drugs undergo transcellular absorption, whereas hydrophilic drugs undergo intercellular penetration. However, the ocular intercellular penetration of hydrophilic drugs is hampered by the presence of tight junctions across the corneal epithelia. This becomes even more challenging in the case of higher molecular weight drugs. Ninety percent of drugs capable of undergoing corneal absorption distribute to the aqueous humour and the anterior eye chamber [15,95]. From there, they again redistribute to the neighbouring ocular tissues including the lens, the ciliary body, the iris, the vitreous, and the posterior retina at variable rates [15]. For example, drug distribution from the aqueous humour to the vitreous occurs at a relatively slow rate due to the retarding effect of the lens. However, it is important to note that drugs in the aqueous humour are subject to melanin binding [6]. In addition, they have very short half-lives (typically 1 h), where they are subject to rapid clearance by the rapid turnover of the aqueous humour, which is continuously formed and is approximately replaced every 100 min before draining into the venous blood circulation through the canal of Schlemm and the trabecular meshwork [90]. Other non-conventional elimination routes including the uveroscleral and uveovortex pathways have also been proposed for the drainage of aqueous humor [92].

Thus, the corneal epithelia, stroma, conjunctival blood and lymphatic clearance represent the main barriers of the anterior eye segment that hamper topical drug absorption to the posterior eye [91].

With respect to the posterior eye segment, there are several barriers that affect drug permeation to the back of the eye including sclera, choroid, Bruch’s membrane, the blood retinal barrier and the retinal pigment epithelium (RPE).

Although the leaky walls and the rich blood supply of the choroid in the posterior eye segment can allow oral as well as injectable drugsto access the choroidal extravascular space, however, further drug distribution from the choroid to the retina is restricted by the blood retinal barrier that will be described later in this section. Furthermore, the choroidal blood flow increases drug drainage to the systemic circulation and aids in the drainage of the aqueous humour through the uveoscleral pathway from the anterior eye chamber [96]. Additionally, complex ocular fluid mechanics including liquid flow from the vitreous to the anterior chamber complicates drug diffusion.

Thus, posterior eye segment tissues including vitreal fluid flow, sclera, choroid, Bruch’s membrane, and the retinal pigment epithelium (RPE), as well as drug clearance through choroidal blood flow and lymphatic drainage represent the main posterior segment absorption barriers that limit drug absorption to the retinal tissues [91].

In addition to the above anatomical barriers, it is important to note the role of the blood ocular barrier. As mentioned earlier, such as the brain, the eye is also very well protected by an efficient restrictive blood ocular barrier system that maintains the eye as a privileged site against xenobiotics in the blood stream. These blood ocular barriers represent the main reason for the failure of orally and systemically administered drugs from reaching therapeutic concentrations in the anterior as well as posterior eye segments. The blood-ocular barrier consists of two main barriers as shown in Figure 4D, the blood aqueous barrier, and the retinal blood barrier. The blood aqueous barrier is composed of two main tissues, (1) the tight junctions in the vascular endothelium of the iris along with the iris blood vessels and, (2) the non-pigmented ciliary epithelium existing in the ciliary body [91]. With respect to the blood retinal barrier, there are two retinal regions in direct interaction with the blood: (1) The retinal vessels region, which is protected by the presence of tight junctions existing between the retinal vessels endothelial cells (the inner blood retinal barrier), and (2) The RPE-choroid interface, which is protected by the presence of tight junctions in the RPE (the outer blood retinal barrier) [97,98].

Successful therapeutic drug delivery depends on the delivery site, tissue barriers, and the type of pharmacological agents involved. Therefore, for optimal retinal drug delivery, it is important to take into account the various ocular barriers encountered (static absorption barriers (corneal, conjunctival, scleral, vitreal), dynamic ocular barriers (choroidal and conjunctival drainage into the systemic circulation and lymphatic clearance), and blood ocular barriers (blood aqueous and blood retinal barriers)). Furthermore, the limited ocular surface area, the long diffusional length to reach the posterior segment, melanin binding, efflux pumps, as well as other physiological factors such as dosage spill-over, nasolacrimal drainage, blinking, as well as removal by tear film and tear mucin also need to be considered [75,77,91].

### 3.2. Pathways of Topical Drug Absorption into the Posterior Eye Segment

Due to the numerous barriers encountered, only upto 1/100,000th the dose of topically applied drugs is able to reach the retina. For this reason, it has always been perceived that topical drugs fail to access retinal tissues [15]. However, over the last couple of decades, several authors have demonstrated the ability of topically applied drugs to reach the posterior eye segment [99,100,101,102,103,104,105]. Thus, the applicability of using topical delivery to deliver drugs to the posterior eye segment tissues is currently being reinvestigated.

**Figure 6:**
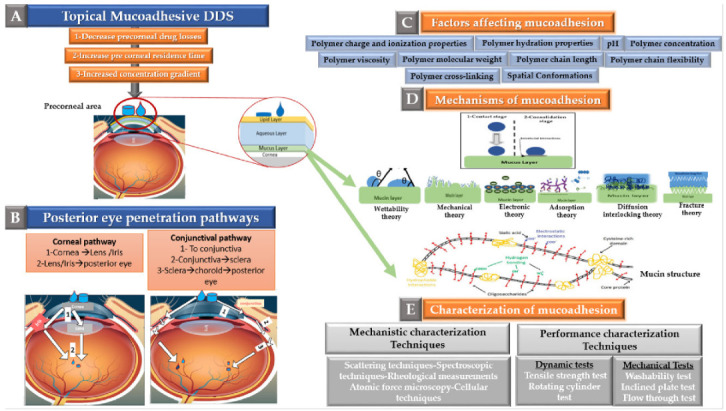
Role of mucoadhesion in promoting topical drug delivery to the posterior eye segment (**A**) Rationale of using mucoadhesion for posterior eye drug delivery, (**B**) Pathways of topical drug permeation to the posterior eye segment, (**C**) Factors affecting mucoadhesion, (**D**) Mechanisms and theories of mucoadhesion, (**E**) Characterization techniques of mucoadhesion (adapted from Sai H.S. Boddu et al. [15], Bentham Science, 2014 and Ankita Garg et al. [106], Innovare Academic Sciences, 2012).

The pathways that have been elucidated to explain topical drug delivery to the posterior segment are illustrated in Figure 6B, and have been thoroughly reviewed by Sai H.S. Boddu et al. [15]. In brief, there are two main pathways for a topically applied eye drop to access the posterior eye segment as follows:

1. The corneal pathway: The corneal pathway has been reported to be the primary route of hydrophobic drugs absorption to the posterior eye segment [95]. Following corneal absorption, drugs can then reach the posterior segment using one of two ways as follows:
  - Following corneal absorption, 90% of the absorbed drugs are distributed to the anterior eye chamber [15,95]. From there, they again redistribute to the neighbouring ocular tissues including the lens, the ciliary body, the vitreous, and the posterior retina [15].
  - The drugs absorbed through the cornea can also undergo lateral diffusion to the sclera, from where they can be distributed to the various ocular tissues including the posterior segment tissues [15].
2. The conjunctival pathway: This pathway is reported to be the major pathway for the absorption of hydrophilic drugs (e.g., Inulin) to the posterior eye segment tissues [95]. In this pathway, the anterior chamber is bypassed and drug distribution occurs predominantly in the uveal tract and vitreous humour. Through the conjunctival pathway, drugs are reported to access the posterior eye tissues through one of the following:
  - Diffusion across the conjunctiva, choroid, and sclera to reach the retina (this is the major pathway) [15].
  - Conjunctival absorbed drugs can also diffuse laterally to the iris, cornea, and ciliary body, i.e., diffuse to the anterior chamber with other intraocular tissue [15].
  - The conjunctival blood vessels can drain drugs to the systemic circulation, from where they distribute to various body organs, including the retina [15].

As the drug’s concentration gradient across the ocular surface plays a key role in deciding the degree of corneal and conjunctival absorption. Therefore, employing mucoadhesion to increase adherence to corneal and conjunctival epithelia, and hence decreasing precorneal drug loss while prolonging drug residence in the tear film, is expected to allow increased delivery to the posterior eye segment as illustrated in Figure 6A. For this reason, mucoadhesion is currently being explored for its potential in promoting non-invasive drug delivery to the posterior eye segment as will be discussed in Section 4.

## 4. Mucoadhesion for Posterior Eye Segment Delivery

Mucoadhesion is a narrower subtype of bioadhesion in which adhesive attractive forces act to attach two surfaces together, one of which is a mucosal membrane [107]. Bioadhesion is a very interesting and widespread phenomena in which adhesive attachment forces are observed in biological systems [108]. In pharmaceutical applications, bioadhesion refers to the approach in which the drug delivery system is designed to adhere to a specific biological surface. If this biological surface is one of the body’s mucosal membranes, this phenomenon is called mucoadhesion [108].

Mucous membranes are widely distributed throughout the body. They represent the moist surfaces which line the body cavities such as the eye, the respiratory tract, the genital tracts, and the gastrointestinal tract, among others. They are made of a layer of connective tissue, whose surface is covered by epithelial tissues, and are rendered moist by mucus secretion. Secretion of mucus occurs on a continuous basis to lubricate, hydrate and protect epithelial surfaces from dirt, pathogens and extraneous toxic material. In addition, it aids gas and nutrients exchange as well as water and electrolyte balance [107,109].

Due to the wide distribution of mucus throughout the human body, drug delivery through several mucosal sites can be optimized and enhanced by exploiting mucoadhesion [110]. Although pharmaceutical interest in mucoadhesion started growing rapidly since the 1980s, earlier reports of using mucoadhesive pharmaceutical formulations dates back to 1947, when Scrivener and Schantz mixed gum tragacanth with dental adhesives in an attempt to improve penicillin delivery through the oral mucosa [111].

The ability of mucoadhesion to attach pharmaceutical drug delivery system to a specific mucosal site allows optimizing delivery through (1) Enabling the application of the drug and restricting its location within the desired absorption window, (2) Prolonging the residence time of the loaded drug at the application site and preventing its rapid clearance and (3) Creating a concentration gradient of the drug at the site of application [108]. All these factors, along with the rich mucosal blood supply in some mucosal tissues, allow the achievement of maximal drug absorption and bioavailability at reduced drug concentrations. This not only reduces drug toxicity and side effects, but also increases patient compliance and adherence, leading to better treatment outcomes and quality of life. Moreover, mucosal administration can serve as an alternative non-invasive economical route for the delivery of sensitive drugs such as proteins and oligonucleotides while bypassing the first pass metabolism and the gastrointestinal tract (GIT) degradation encountered with the oral route [108].

### 4.1. Theory of Mucoadhesion

The mucus structure allows for the occurrence of different types of interactions with mucoadhesive materials. Mucus is a viscoelastic gel layer that lines the mucosal tissues and is highly hydrated due to its high water content (90–98%), and contains 0.2–5% *w*/*w* mucins, 0.5% *w*/*v* proteins, as well as salts, lipids, bacteria, DNA, cells, and cellular debris [18]. Mucus characteristics can vary significantly in different body locations. Therefore, mucoadhesion intended for ophthalmic delivery should take into consideration route specific considerations such as eye’s mucus features, as well as ophthalmic tissues anatomy and physiology. Ocular mucus supports the adhesion of the tear film to the eye surface, thus enabling it to perform its crucial defensive, nutritional, mechanical, as well as optical functions [25]. It has been reported that the tear film coverage is essential for corneal smoothness as well as structural maintenance, integrity, and lubrication. Furthermore, most of the cornea’s oxygen supply, which arises from direct air exposure, is absorbed by the tear film before diffusing to corneal tissues. Ocular mucus has been explored as a non-invasive delivery alternative to deliver drugs to the anterior and posterior eye segments, as will be discussed in Section 5 and Section 6. Mucin is the most important structural component of mucus, and is responsible for its characteristic adhesive and cohesive gel-like structure.

#### Ocular Mucins

**Figure 7:**
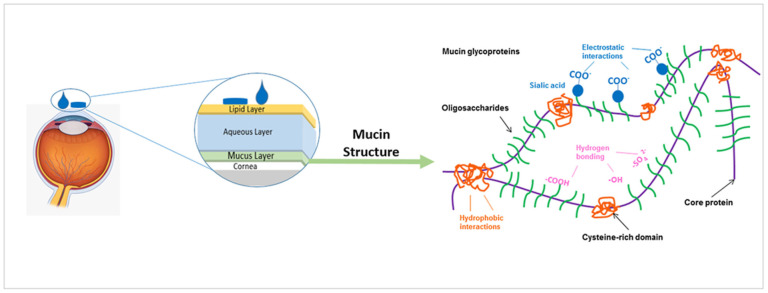
Mucin Structure. Adapted from Xiaoyun Yang et al. [114], PLoS, 2012.

Mucin is made up of glycoproteins, which consist of a protein core covalently linked to carbohydrate side chains through *O*-glycosidic linkages as illustrated in Figure 7 [18,107]. Depending on the type of mucin involved, mucus exists either as a membrane bound gel layer adherent to the surface of mucosal tissue, or as a luminal soluble or secreted form [107]. Membrane-bound mucins play crucial roles in cell signalling and cellular protection and function to form the glycocalyx, which links the secreted gel layer to the cell surface. The soluble or secreted mucins on the other hand are characterized by their high molecular weight, gel forming properties, and high *O*-linked carbohydrates ratio and are secreted by goblet cells and submucosal glands. Mucins properties are largely dependent on their concentration and the type of associated materials existing in their local environment and thus, could be variable in different parts of the body. The mucus properties at different mucosal sites has been well documented by Jasmim Leal et al. [112]. For example, extensive mucin glycosylation is associated with increased stiffness, however, mucins’ basic features remain controlled by their physicochemical properties including their high molecular weight, hydrophilicity, and negative surface charge associated with the sulfate or sialic acid groups as shown in Figure 7 below [109].

An excellent overview of ocular mucins and their effects on tear film properties has been described by Georgi A. Georgiev et al. [113]. Ocular mucus demonstrates the presence of both transmembrane mucins (MUC1, MUC4, MUC13, MUC15, MUC16, and MUC17), as well as secretory mucins (MUC2, MUC5AC, and MUC7) [109]. The presence of both mucin types serves vital roles such as the modulation of the tear film viscoelasticity and surface tension properties, as well as increasing the wetting of the ocular surface glycocalyx. The mucus secreting goblet cells present in the conjunctiva are responsible for the production of the ocular mucus. This mucus once secreted then spreads and stretches by the wiping upper lid movement to cover the corneal surface, which has no mucus secreting goblet cells [109].

Interactions occurring at the mucus interface take place through different types of chemical bonds including ionic bonds, covalent bonds, hydrogen bonds, Van der Waals bonds, and hydrophobic bonds [107]. Several theories have been proposed to explain the complex mucoadhesion phenomenon occurring at the mucin/mucoadhesive formulations interface. These theories are summarized and illustrated in Table 2, and include the wetting theory, the electronic theory, the adsorption theory, the fracture theory, the mechanical theory, and the diffusion interlocking theory [107,115]. However, the basic mechanistic description of mucoadhesion is based on two main stages as illustrated in Figure 6D, (1) The contact stage:-in which the mucoadhesive material obtains into intimate contact with the mucous surface, and (2) The consolidation stage:-in which the bioadhesive material starts to interpenetrate into the mucosal layer initiating various physicochemical interactions that act to consolidate and strengthen the formed interfacial adhesive joint [110]. It has been proposed that for formulations undergoing higher physical stresses, as is the case in ocular delivery, the second consolidation stage is of utmost importance as it prevents the formulations’ dislodgement and aids in their fixation. An excellent overview of the detailed mucoadhesion theories and its underlying mechanisms has been well documented by Smart J.D. [107].

### 4.2. Factors Affecting Mucoadhesion

Among the various materials employed in drug delivery, polymers have been the most widely explored for mucoadhesion. As shown above, polymer physicochemical properties play a crucial role in deciding the strength of the mucoadhesive joint formed with the mucosal surface. Thus, in addition to biocompatibility, safety, and biodegradability, several important polymer characteristics need to be taken into account. The polymer characteristics that affect mucoadhesion have been reviewed extensively [18,110], and include properties that influence the polymer’s ability to interact efficiently with the mucosal surface mucins such as polymer molecular weight, charge, degree of cross-linking, chain length, flexibility and spatial conformations, concentration, hydration properties, in solution viscosity, as well as pH of the involved medium as shown in Figure 6C.

### 4.3. Characterization of Mucoadhesion

Many characterization techniques have been described in literature for the assessment of mucoadhesive drug delivery systems. Mucoadhesion characterization techniques could be classified in several ways. An excellent overview of the different mucoadhesion characterization techniques and their classification has been well documented by Alan R Mackie et al. [109]. Some reports classify mucoadhesion characterization techniques according to the physical phenomena involved in measurement. Others classify them based on the type of dosage form that can be tested. Additionally, classification into molecular scale and macro scale testing has been reported [109]. In this review, we will classify mucoadhesion characterization methods into (1) Mechanistic methods—which describe interaction mechanisms at the level of the mucoadhesive joints, and (2) Performance or functionality methods—which describe the actual mucoadhesive performance of the whole drug delivery system.

#### 4.3.1. Mechanistic Characterization Techniques

A wide range of characterization techniques have been developed to give a useful insight into the interaction mechanisms happening at the level of the mucoadhesive joints. For mechanistic understanding of mucoadhesion, detailed understanding of mucin’s structure is crucial. Several high resolution scattering techniques including small angle neutron scattering (SANS), small angle X-ray scattering (SAXS), static light scattering (SLS) and dynamic light scattering (DLS) have been reported to contribute to the structural understanding of mucin, as well as the properties that contribute to mucoadhesive interactions.

To study interfacial mucoadhesive interactions, various spectroscopic techniques including ^1^H and/or ^13^C nuclear magnetic resonance (NMR) as well as Fourier transform infrared spectral analysis (ATR–FTIR) have been used to analyse the affinity of mucoadhesive polymers to mucins. Spectroscopic analysis is an in vitro mechanistic testing approach has been used to describe and characterize mucoadhesion on the molecular scale. It is very useful to investigate polymer mucus interactions. NMR analysis has been reported to be beneficial for characterizing mucin glycoprotein interactions with formulations. This is attributed to the avoidance of sample pre-treatment or derivatisations, thus avoiding structural alterations of the analysed materials. ATR–FTIR has also been used to evaluate and understand the interactions occurring at the interfacial level between mucus and mucoadhesive formulations [109].

Another widely exploited tool that evaluates mucus/mucoadhesive polymers mechanistic interactions is rheological analysis. Unlike spectroscopic techniques that evaluate molecular level interactions, rheology evaluates interfacial interactions on the macromolecular level. As mentioned before, mucus is a biological macromolecule based material with viscoelastic gel-like behaviour. In solution mixing of mucus with another macromolecule (mucoadhesive polymer) is used to allow for the occurrence of interactions. These interactions induce changes in the flow behaviour of the mixture in comparison to those of each sole component. Thus, rheological assessment has been widely exploited to give useful insight into macromolecular mucoadhesive interfacial interactions.

Atomic force microscopy (AFM) is another useful and interesting mechanistic approach to study mucoadhesion. AFM stands out in its ability to image the quantity and conformation of adhering materials, while allows for sensitive adhesion measurements using force spectroscopy. In AFM, the tested particles are glued to the AFM cantilever, which is then introduced vertically into the sample surface. The measurement of cantilever deflections during the approach of the measuring probe and its retraction away from the sample’s surface reflects the strength of the mucoadhesive bonds involved. AFM is advantageous in allowing sensitive force measurements, however, it remains a time consuming technique especially in heterogeneous samples where several areas of the sample need to be considered independently.

Interactions between mucin and mucoadhesive materials can also be investigated using in vitro cell culture techniques. Cellular methods give good insight into interaction with mucosal tissues; however, the dependence of this approach on biological mucosal tissues is associated with several problems including limited availability as well as high variability [109].

#### 4.3.2. Performance Characterization Techniques

Functionality characterization techniques give valuable information into the actual mucoadhesive performance of drug delivery systems. Some performance tests depend on the mechanical evaluation of formulae and some depend on their dynamic testing.

The principle of mechanical performance tests is based on the measurement of the force required to detach the tested mucoadhesive formulation from mucosal surfaces. The most common tests under this category are tensile strength testing and the rotational cylinder testing method [109].

Tensile strength evaluation of various mucoadhesive formulations including tablets, fibres, hydrogels, and films can be performed using texture analysers. In the experimental set-up, the tested formulation is attached to a probe that brings the formulation in close contact with the mucosal surface. After sufficient incubation, mucoadhesion strength is estimated based on the force required to detach the formulation from mucus [109].

In the rotating cylinder test method, the tested formulation is attached to a disc that is allowed to adhere to a mucus-covered cylinder. This cylinder is then submerged and rotated in the testing buffer. The time taken by the formulation disks to dislodge or dissolve is measured and reflects the mucoadhesive strength of the examined formulation [115].

On the other hand, in dynamic based testing of mucoadhesive performance, the tested formulations are assessed based on their behaviour in response to mimicked physiological clearance mechanisms as in the washability test, inclined plate test, and flow through test [109,117].

In the washability test, in addition to testing the mucoadhesive character, the ability of the system to stay in contact with the mucosal membrane under the washing effect of physiological fluids is also assessed. This test can be easily performed using Franz diffusion cells with some slight modifications [117]. The idea of the test is to optimize the buffer streaming conditions through the sample in such a way that mimics the washing away mechanisms encountered. For example, the tendency of mucoadhesive ophthalmic formulations to withstand removal by lachrymal fluids can be assessed using a thermostated buffer solution maintained at the tear fluid physiological ophthalmic pH (7.4) and fluxed at a rate mimicking that of eye fluids [117].

In the inclined plate method, an accurately weighed amount of a liquid or semisolid formulation is placed on a horizontal mucin coated substrate holder or plate. When this plate is inclined at a predetermined angle, the quantity of the formulation falling off is recorded as a function of time using a microbalance until reaching the plateau. The percentage of the sample that adhered to mucus is then calculated using the difference between the initial loaded sample amount and the amount that fell off the plate [109,117,118].

Another dynamic based performance test is the flow through test. In this test, fluorescent-labelled colloidal particles are applied as droplets at the centre of a hydrophilic membrane that has been previously soaked in an aqueous mucin solution for 2 h. A peristaltic pump is then used to pass a washing solution through the mucin-covered membrane as a rough simulation of the tear fluid flow. The number of particles that resist washing and remain adherent to the mucin coated membrane are then counted using microscopy or fluorescence spectroscopy. In an attempt to study mucoadhesion under simulated tear fluid flow, Choy et al. used this technique for the assessment of the mucoadhesion of poly (lactic-*co*-glycolic acid) (PLGA) and poly (ethylene glycol) (PEG) based microparticles that have been engineered for ophthalmic mucosal delivery. In their study, the authors used rhodamine labelling to quantify the amount of microparticles that remained adherent to the mucin coated membrane and compare between the different tested microparticles. In comparison to PLGA microparticles, in which only 29% of the applied particles remained adherent to mucin, up to 53% of the PEG/PLGA microparticles in which PEG has been added to improve mucoadhesion remained adherent to mucin [119].

Additionally, Chaiyasan et al. reported the use of the same technique to study the mucoadhesive behaviour of chitosan-dextran sulfate nanoparticles that have been designed for delivery to the ocular surface. However, in this study, the authors used fluorescein isothiocyanate labelling instead, and studied the effect of in vivo shear stresses using a peristaltic pump that maintained a steady stream of a washing saline solution [120].

Performance characterization techniques can be very helpful during the design and optimization of mucoadhesive DDs, where they can provide useful insight on the relative ability of different tested formulae to remain adherent to the mucosal surface in response to simulated physiological forces that act to detach topical formulations from mucosal surfaces.

### 4.4. Considerations for Mucoadhesion in Ocular Drug Delivery

In comparison to other mucosal routes, ocular mucosal delivery is far more challenging. The hypersensitivity of ocular tissues requires the development of non-irritant drug delivery systems; to avoid excessive blinking and lacrimation induced formulation removal. Special attention should also be paid to restrict the formulation location to the conjunctival mucus rather than the loosely attached mucus within the corneal area. Binding of a formulation to the corneal mucus does not only result in inefficient mucoadhesion, but also interferes with vision. It is important to denote that any abnormalities in the tear film’s coverage which is essential for corneal smoothness will affect the cornea’s transparency and contours required for proper vision [90]. Thus, it is of utmost importance to select a comfortable ocular drug delivery systems design that neither interferes with the tear film properties, nor impacts vision adversely upon topical application.

Furthermore, ocular mucus, being of very fine thickness, necessitates the selection of mucoadhesive polymers that are capable of creating strong adhesive joints even at the low mucosal thickness encountered. Thus, the use of strong and well characterized mucoadhesives that are efficient, non-irritant and safe is of utmost importance for ocular mucosal delivery. In addition, care should be taken to ensure complete drug release and absorption before mucin turnover, which occurs every 15–20 h [109]. As mentioned earlier, the high blood supply of mucosal tissues makes them highly favourable for enhanced drug absorption and bioavailability. However, one distinct feature of ocular mucosa is that it does not only lack blood supply, but is also lined by the cornea which is itself a permeability barrier, and thus prolonged exposure is required for enhanced absorption. Another main challenge facing ocular mucosal delivery is the small ocular surface area and the small tear volume (7 μL). This imposes restrictions on the formulation volume or surface area (in case of solid dosage forms) that can be administered. Thus, restricting drug loading capability, limiting applicability to low dose potent drugs [109].

With respect to mucoadhesion for posterior eye delivery, several mucoadhesive polymers including chitosan, hyalouronic acid, polyvinyl alcohol, and alginates have been investigated as will be discussed in Section 5 and Section 6. Among these polymers, the cationic polymer chitosan has been the most explored for promoting topical drug delivery to the posterior eye segment as discussed in more detail in Section 5.

## 5. Chitosan-Based Mucosal Delivery

**Figure 8:**
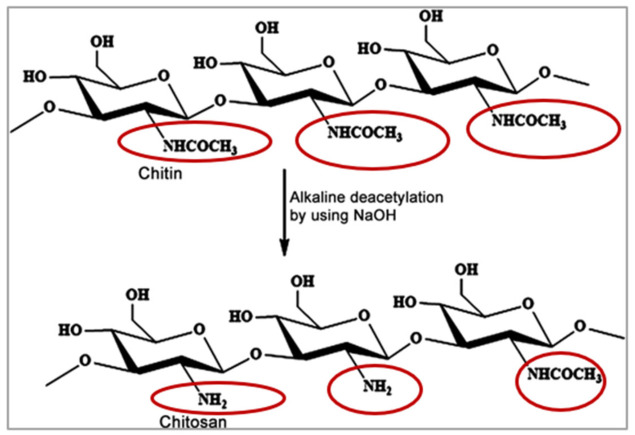
Structure of chitosan and its preparation from chitin. Adapted from Yassmin G. Saleh et al. [121], Faculty of Women for Arts, Science and Education ASU, 2016.

Among the numerous polysaccharides available in nature, chitin is the second most abundant following cellulose [17]. Chitin is a major constituent of marine invertebrates, and is produced in billions of tons every year from insects, crustaceans, fungi, and molluscs among many other organisms. Structurally, chitin is a linear polysaccharide that is formed of (1,4)-linked *N*-acetyl-d-glucosamine units as illustrated in Figure 8 [16]. CS is obtained from chitin mostly using partial alkaline deacetylation, and is one of the most investigated polymers in drug delivery [19]. The process of alkaline deacetylation of chitin yields *N*-acetyl-d glucosamine units linked to d-glucosamine units via 1,4-glycosidic linkages as shown in Figure 8. Both chitosan’s quality and bioactivity are defined by its molecular weight, as well as its degree of deacetylation [16,17].

Among the most important features of CS that make it attractive and widely explored in drug delivery are its biocompatibility and biodegradability. Its biodegradability is attributed to its structural similarity to the physiological glycosaminoglycans. It is thought to undergo enzymatic degradation by lysozymes at the following linkages: (1) the glucosamine–glucosamine linkage, (2) *N*-acetyl-glucosamine-*N*-acetly glucosamine linkage, and (3) glucosamine-*N*-acetyl glucosamine linkages. In addition to degradation by lysozyme, chitinases and acid hydrolysis are also thought to contribute to degradation of CS [16,17]. In addition, CS has gained FDA approval for the development of wound dressings due its regenerative features and mucoadhesive properties, and is also used for weight control in some marketed dietary supplements [17].

As mentioned earlier, chitosan’s molecular weight as well as degree of deacetylation affect its quality and activity. Chitosan’s pKa is ~6.6, and when its degree of deacetylation approaches ~50%, it becomes soluble in acidic media and its amino groups protonate giving CS its characteristic cationic nature [17,122]. Chitosan’s cationic charge allows its exploitation in many drug delivery applications, and is responsible for its potential in gene delivery, vaccine adjuvant properties, antimicrobial activities, as well as formation of ionic interactions with a vast number of negatively charged polymers [16,17,18,19]. In addition, the electrostatic interactions between chitosan’s positive charges and the negatively charged sialic acid residues of mucus account for its mucoadhesive potential, which has been widely investigated to increase residence time, and achieve sustained drug release profiles [16,17,18,19].

In addition to its mucoadhesive properties, CS also has penetration enhancement activity [19]. This activity is again attributed to its positive charge, which mediates its interactions with the involved cellular membranes. On interacting with a certain cell membrane, CS acts to modify its intercellular tight junction proteins. These modifications lead to the opening of these intercellular tight junctions. Direct evidence of the involvement of tight junctions opening in the permeation enhancement of CS has been obtained from both Caco-2 cells and in vivo mice studies, where tight junctions opening is reported to be associated with a decrease in TER, and an increase in paracellular permeability, thus creating permeation pathways for drugs to traverse these mucosal cells [16,17].

The dual ability of CS to increase drug residence time at their site of administration through its mucoadhesive effects, while creating permeation pathways for drugs through its permeation enhancing effect is the reason for its wide exploration in ophthalmic drug delivery, where precorneal drug loss and topical drug absorption are specially problematic. Thus, most papers investigating the potential of mucoadhesion in promoting posterior segment eye diseases employed CS for its outstanding ability to act as both a mucoadhesive as well as permeation enhancer, as will be discussed in this section.

To utilize chitosan-based strategies for promoting posterior segment eye delivery, it is crucial to take note of the critical quality attributes of chitosan that affect its mucoadhesive and permeation enhancement activities. As the mucoadhesion potential of chitosan is based on the electrostatic interaction between its positively charged amino groups and the negative charges of sialic acid of mucus, therefore, using high molecular weight chitosan grades that have a high degree of deacetylation (higher number of free amino groups) show better mucoadhesion potential. Another important factor to consider is the degree of crosslinking, the higher the degree of crosslinking, the less the mucoadhesive interactions. In addition, it is important to take note of the medium’s pH, as optimal mucoadhesion potential of chitosan is observed in acidic pH media where the polymer is soluble, and where its amino groups become protonated for optimal interaction with mucins [16].

With respect to the permeation enhancement activity of chitosan, it is also important to note the effect of several polymer characteristics namely chitosan’s molecular weight, degree of deacetylation, as well as salt form, with the degree of deacetylation being the most prominent. Chitosan’s degree of deacetylation is reported to significantly influence its permeation enhancement effect. This has been extensively reviewed in the literature [17], and is explained by the role of chitosan’s positive charges in mediating its interactions with the cell membrane during permeation enhancement. Thus, the presence of a higher number of free amino groups in high molecular weight chitosan grades that have higher degrees of deacetylation increases tissue permeation enhancement and vice versa. Another critical factor to consider is the pH of the medium. An acidic environment that maintains chitosan in its soluble and positively charged form is critical for keeping the permeation enhancement activity of chitosan and vice versa. At pH values exceeding chitosan’s pKa (6.5–6.6), chitosan’s aqueous solubility and hence charge density decreases, resulting in its precipitation and loss of activity [17].

Thus, while studying the potential of chitosan in promoting posterior segment eye delivery, it is of crucial importance to consider not only chitosan’s grade, molecular weight, and degree of deacetylation, but also the pH of the ocular surface to which the drug delivery system will be applied. Despite having a high buffer capacity, the eye’s pH is normally neutral, and thus chitosan’s mucoadhesive and permeation enhancement activities are expected to be greatly compromised when applied topically on the ocular surface. A potential solution to this is to use slightly acidic formulations that promote Chitosan’s mucoadhesive interactions. However, these formulations will increase reflex blinking and lachrymation, which will in turn lead to increased precorneal clearance and dosage form removal from the ocular surface.

In this section, chitosan-based approaches that have been investigated in literature to promote topical drug absorption to the posterior eye segment are reviewed.

### 5.1. Chitosan Nanoparticles

Several methods have been reported for the preparation of CS nanoparticles [123]. These methods include: (1) ionic gelation [124], (2) polyelectrolyte complexation [125], (3) complex coacervation [126], (4) drug complexation [127], (5) emulsification solvent evaporation [128], and (6) self-assembly [129]. A detailed overview of biofabrication considerations for the development of CS based nanosytems for ophthalmic drug delivery has been reported by Riddhi Vichare et al. [130].

In contrast to the conventional nanoparticles production techniques, ionic complexation based techniques usually have the advantage of avoiding the use of organic solvents. However, it is important to denote that chitosan being a basic polymer, it is water insoluble (pH = 7), and requires acidic media for its solubilisation and processing [130]. Thus, one of the most critical factors to consider during the formation of CS nanoparticles utilizing ionic complexation is the processing pH, and its possible destabilizing effect on the encapsulated drug’s stability [130].

As mentioned earlier, several critical quality attributes of CS such as (1) degree of deacetylation, (2) degree of polymerization, as well as (3) molecular weight need to be carefully considered during the exploration of its potential in drug delivery [16,17,130]. Chitosan’s degree of deacetylation can be accurately determined using a wide variety of available techniques such as UV-Vis spectroscopy, infrared spectroscopy, ^1^H-NMR and elemental analysis. Chitosan’s degree of deacetylation is not only critical for its significant impact on mucoadhesion and permeation enhancement as previously discussed, but because it also affects crystallinity, solubility, viscosity, biocompatibility, and biodegradability, and hence can have a massive impact on the process of chitosan nanoparticles production as well as the physicochemical properties of the resulting chitosan-based nanosystems [130]. With respect to the determination of chitosan’s molecular weight and molecular weight distribution, several techniques are available and can be used such as intrinsic viscosity determination, size exclusion chromatography, gel permeation chromatography, as well as static light scattering [130].

CS based nanoparticles have been recently investigated for posterior eye delivery by Beatriz Silva et al. [131]. In their work, the authors developed erythropoietin loaded chitosan-hyalouronic acid nanoparticles prepared by ionotropic gelation. Six different grades of hyalouronic acid were compared, and the best hyalouronic acid grade was selected based on its strength of mucoadhesive binding. Upon optimizing nanoparticles production, optimal nanoparticles were produced at the 1:1 CS:HA mass ratio yielding nanoparticles of size ≤300 nm, PDI values ranging from 0.167–0.539, and having an average zeta potential of around +30 mV. With the different HA grades tested, %EE values ranging from 35.2 ± 0.5 to 39.9 ± 0.6 were obtained, and the % loading capacities obtained ranged from16.0 ± 0.2 to 18.1 ± 0.3. The authors then evaluated the nanoparticles for their in vitro drug release properties, after which their mucoadhesive binding to mucin was tested using rheology as well as zeta potential determination. Furthermore, the cytotoxicity of the nanoparticles were tested on the ARPE-19 and HaCaT cell lines, where they were demonstrated to be non-toxic. The nanoparticles were then assessed for ex vivo permeation through fresh porcine corneas, scleras and conjunctivas, where the permeated erythropoietin amount was quantified using ELISA, along with immunohistochemistry to check its presence in the membranes. Among the three tested tissues, the conjunctiva was the most permeable to hematopoeitin loaded chitosan hyalouronic acid nanoparticles. However, the authors pointed to the importance of carrying out further studies to confirm that erythropoietin structure and stability have not been adversely affected by the acidic conditions used in the preparation of CS hyalouronic acid nanoparticles. In addition, further in vivo pharmacokinetic and pharmacodynamics studies are needed to assess the effect of the various ocular barriers including blinking, tear film clearance, conjunctival/ choroidal drainage to systemic circulation, etc.

### 5.2. Chitosan Coated Drug Delivery Systems

**Figure 9:**
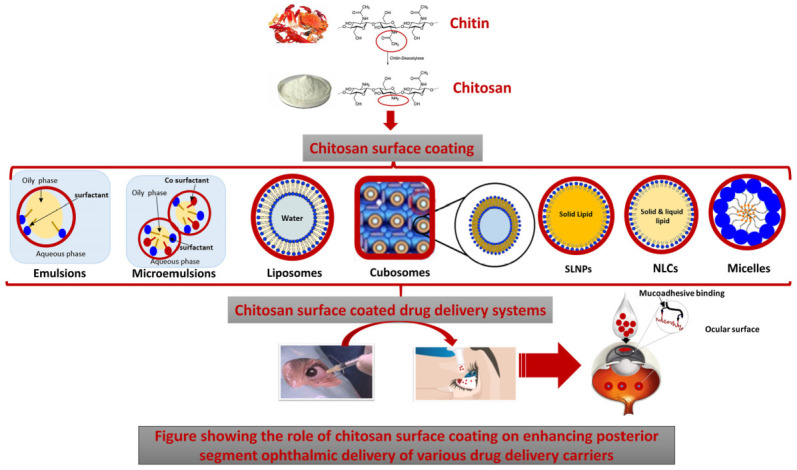
Role of chitosan surface coating on promoting posterior segment drug delivery of various nano-sized drug delivery carriers.

Another investigated approach utilizes CS surface coating of a wide range of drug delivery systems for promoting topical drug delivery to the posterior eye segment as illustrated in Figure 9. The various CS coated drug DDSs that have been explored for PSEDs are reviewed in this section as summarized in Table 3. As mentioned above, it is important to take note of CS properties that affect its interaction with mucus as well as formulation characteristics. Similarly, it is important to consider the impact of CS properties on its surface coating efficiency. These factors have been thoroughly reviewed by Bugnicourt L. et al., and serve as a useful guide for the development of chitosan coated DDSs [132].

#### 5.2.1. Chitosan Coated Emulsions

##### Emulsions

An emulsion is a system that consists of a mixture of two or more immiscible liquids, usually oil and water, where one liquid is dispersed as fine droplets throughout the other using surface active agents and energy input [133]. Emulsions possess many merits for ophthalmic drug delivery such as (1) Allowing the control of drug release, (2) Increasing bioavailability of incorporated drugs, (3) Protection of labile drugs, (4) Having modifiable viscosity which could be tailored for prolonging drug residence at the ocular surface, (5) Increasing the tear fluid viscosity upon application, which increases the residence time of the formulation in the tears, and (6) They contain surfactants which can increase the corneal epithelial cells permeability to enhance corneal drug penetration and on the incorporation of cationic surfactants, the emulsion droplets possess positive charges that can interact with the negatively charged corneal epithelium to increase residence time [133]. Moreover, in comparison to other drug carriers, they are biocompatible, biodegradable, physically stable, and easier to develop and manufacture [134].

Due to their ability to improve ocular bioavailability while maintaining patient comfort and acceptability, oil in water lipid emulsions have been commercialized for topical ophthalmic drug delivery. The first commercialised lipid emulsion for ophthalmic delivery was the 0.05% cyclosporine A anionic lipid emulsion that was developed by Allergan (Restasis^®^) for the management of chronic dry eye disease. Following the release of Restasis^®^, several cationic emulsions including Cationorm^®^ and Novasorb^®^ have also been introduced for curing dry eye syndrome and for the efficient delivery of drugs for anterior segment eye diseases [135].

To exploit the advantages of lipid emulsions in posterior segment eye delivery, Ying et al. investigated the potential of CS coated lipid emulsions for posterior eye drug delivery via eye drops. Coumarin-6, being a fluorescence marker, was used as a model drug for the in vivo assessment of retinal drug delivery in mice. To favour posterior segment eye delivery, the authors attempted to develop several coumarin-6 loaded lipid emulsions of droplet sizes ranging from 75.95 nm to 160.3 nm using high-pressure homogenization. They studied the effects of oil phase composition, emulsifier type, charge supplement, and surface coating on retinal drug delivery. The authors reported that the surface charge properties of the lipid emulsions were the limiting factor in deciding retinal delivery, i.e., in comparison to the formulation characteristics (properties of the inner oily phase and the type of phospholipid emulsifier) which did not impact the efficiency of drug delivery to the retina. Surface modification using a positive charge inducer and a functional polymer such as CS that could allow for electrostatic interaction with eyes had a significant effect in promoting drug delivery to the posterior eye segment [135].

##### Microemulsions (MEs)

MEs are isotropic and thermodynamically stable clear or translucent colloidal systems of water, oil, surfactant, and co-surfactant with droplet sizes ranging from 5 to 200 nm [133,136]. Adding to their possession of all the advantages of emulsions in ocular delivery, their extremely small droplet size ranges provide the additional advantages of (1) Reducing blurriness of vision upon application, (2) Enhancing ocular drug adsorption and retention time, (3) Enhancing ocular permeability and penetration, (4) Enabling higher control of drug release kinetics. Furthermore, they have superior stability and are easy to produce as drug carriers that can incorporate heat sensitive drugs at higher shelf lives [133].

The use of CS for coating MEs for enhancing posterior segment eye delivery has been evaluated [137]. In their study, Raval et al. developed triamcinolone acetonide (TA) loaded MEs for topical delivery into the posterior eye segment while comparing CS and butter oil as permeation enhancers. The investigated MEs were characterized for their physicochemical characteristics, morphology, in vitro corneal permeation using rabbit corneal cells, and ex vivo permeation in goat cornea. In addition, the authors reported the use of histopathology and fluorescence intensity measurements for the in vivo evaluation of MEs in Sprague Dawley rats. In these studies, the fluorescent marker Coumarin-6 was used instead of TA, and its fluorescence intensity was compared in the different formulations. In comparison to coumarin-6 loaded MEs, both CS as well as butter oil based coumarin-6 MEs showed 4–5 times higher fluorescence intensity, highlighting their roles in promoting posterior segment ophthalmic delivery. The authors attributed the increase observed with CS based MEs to the increased retention and subsequent permeation which results from the electrostatic interaction between the positively charged protonated amino groups of CS and the negatively charged corneal mucin. Furthermore, the role of CS in loosening the tight junctions along with the fluidic nature of MEs was also reported to contribute to the higher permeation of CS based MEs to the posterior eye segment [137].

#### 5.2.2. Chitosan Coated Liposomes

Liposomes are vesicular systems which are made up of a central aqueous core that is enclosed by a phospholipid bilayer. Their particle size ranges can vary from 10 nm to 1 μm or more. According to their structure, liposomes can be classified into (1) unilamellar vesicles (ULVs): where the central aqueous core is surrounded by a single lipid bilayer and, (2) multilamellar vesicles (MLVs) in which the central aqueous core is surrounded by more than one lipid bilayer, each separated by an aqueous compartment. According to their vesicular size, ULVs can be classified into small unilamellar vesicles, large unilamellar vesicles, and giant unilamellar vesicles [138].

Liposomes are not only biocompatible, biodegradable and nontoxic, but their vesicular structure allows them to incorporate hydrophilic as well as lipophilic drugs, where the hydrophilic drugs can be dissolved in their aqueous core and the lipophilic drugs can be solubilized within their phospholipid bilayers structure. In addition, they are flexible to formulate into different sizes and are easily modifiable allowing the incorporation of targeting ligands. Due to their favourable characteristics, liposomes have been extensively investigated for effective drug as well as vaccine delivery through the different routes of administration [139,140,141].

In ophthalmic drug delivery, liposomes have been shown to be able to come into close contact with both the cornea and the conjunctiva for a prolonged period of time leading to enhanced corneal permeability of hydrophilic as well as hydrophobic drugs. Furthermore, they can be easily formulated into different dosage forms that suit ocular delivery such as eye drops, ointments, gels, etc. [138].

The potential of topically applied liposomes for posterior segment eye delivery was first investigated by Hironaka et al. [142]. Altamirano-Vallejo et al. also reported the feasibility of drug delivery into the vitreal cavity and retina using topically applied triamcinolone acetonide (TA) loaded liposomes [143]. To investigate the effect of physicochemical properties of nanocarrier systems on retinal drug delivery via eye drop administration, Inokuchi et al. compared the intraocular behaviour of coumarin-6 loaded lipid emulsions, fluorescein isothiocyanate isomer (FITC) labeled polystyrene particles, and liposomes of different particle sizes, zeta potentials, cholesterol content, and lamellarity in mice, rabbits, and monkeys [144].

In an attempt to enhance liposomal delivery to the posterior eye segment through mucoadhesion, Khalil et al. reported the preparation of chitosan coated liposomes (CCLs) for the topical delivery of triamcinolone acetonide into the posterior eye segment tissues. The TA-loaded liposomes were prepared using thin hydration method, and then to impart mucoadhesiveness to the liposomal surface, 0.1%, 0.2%, and 0.3% chitosan solutions were investigated for coating the liposomal surface to render it positively charged, thus favouring interaction with the negatively charged mucin surface. Successful liposomal coating by chitosan was confirmed by the increased particle size as well as a shift in Zeta potential from −31.8 mV in uncoated liposomes to +14 mV, +20 mV, and +41 mV in 0.1%, 0.2%, and 0.3% CCLS, respectively. In comparison to uncoated liposomes (EE = 63%), CCLs showed higher encapsulation efficiency values for TA (68% at 0.2% Cs coating and 74% at 0.3% Cs coating). CCLs also displayed higher colloidal stability as inferred from the high positive surface charge imparted at 0.3% chitosan (Cs) coating. The efficiency of CCLs in posterior eye segment delivery was assessed in Choroidal neovascularization (CNV) rat models, where significant TA amounts were detected by HPLC analysis in the eye and the vitreous for up to fifteen days after treatment with CCLs. This indicates that Cs coating in addition to increasing the mucoadhesion of CCLs to corneal epithelium, allowed for the successful passage through the mucus barrier of the anterior eye segment and induced transient opening of tight junctions which resulted in increased permeability and, hence, bioavailability of liposomes to the posterior segment [105].

In another study by Li et al., CCLs were investigated for the delivery of TA into the posterior eye segment for the treatment of macular oedema. TA was loaded into liposomes with %EE = 84.04 ± 3.89 using the calcium acetate gradient method. Upon coating the liposomes with 0.5% Cs solution, the %EE of the loaded TA increased to 90.66 ± 3.21. Chitosan surface coating was reflected in an increase in particle size from 108.48 ± 5.59 nm to 135.46 ± 4.49 nm as well as a shift of zeta potential from −10.17 ± 1.71 mV in uncoated liposomes to 7.98 ± 3.21 mV in coated liposomes. The TA loaded CCLs were physically stable and displayed a sustained release profile and did not show significant toxicity on cornea, conjunctiva, and retina. The authors used a Heidelberg Spectralis Optical Coherence Tomography (OCT) system (Heidelberg Engineering, Heidelberg, Germany) for studying relative fluorescence intensity and in cellular uptake experiments. CCLs showed higher transduction efficiency into corneal epithelium HCEC and the retinal pigment epithelium ARPE-19 in comparison to uncoated TA liposomes [145].

In continuation of this work, the same group carried out another detailed study to evaluate CCLs penetration into the posterior eye segment, its therapeutic efficacy in macular edema treatment and safety both In vitro and in vivo [146]. For the evaluation of CCLs permeability and transport to the posterior segment, the fluorescent dye coumarin 6 was used to tag CCLs. Based on the detection of coumarin fluorescence, the penetration and permeability of the fluorescent tagged CCLs was then tested both in vitro and in vivo.

In the in vitro cellular uptake studies, both the corneal epithelium (HCEC) and the retinal pigment epithelium (ARPE-19) demonstrated marked cellular uptake and internalization of the fluorescent-tagged CCLs. This was attributed by the authors to the bonding between the positive charges of the CCLS surface and the negative charges of the cell membrane proteoglycans leading to cytoskeleton rearrangement followed by ruffle formation and subsequent uptake by micropinocytosis which is the major route of cellular entry by positively charged particles.

For the assessment of in vivo penetration of CCLs through ocular barriers, the fluorescently tagged CCLs eye drops were topically applied to the cornea of the examined rodents with OCT images taken every 30 s. At zero time, a portion of fluorescent tagged CCLs was observed on the corneal surface, after 6 min, it appeared in the anterior chamber, and at 10 min, the fluorescent tagged CCLs were detected in the vitreous body and the surface of the retina suggesting the ability of CCLs eye drops to effectively overcome the ocular biological barriers to reach the posterior eye segment.

The preclinical therapeutic efficacy of TA loaded CCLs was then evaluated in vivo using laser induced retinal edema rodent models [136]. In addition to showing significant permeability across the ocular barriers and reaching the retina and choroid, TA-CCLs displayed successful remission of retinal edema in 10 days with histopathology studies revealing almost normal architecture of the retina. Adding to the ability of liposomes to decrease precorneal clearance, the use of the highly mucoadhesive Cs coating led to improved corneal or/and conjunctival adhesion and retention. This prolonged retention, in addition to the penetration enhancing properties of the chitosan coating led to enhanced absorption of CCLs and its cargo through the cornea and conjunctiva sclera. The safety of TA-CCLs was tested in vitro using HCEC and ARPE-19 cells for cytotoxicity studies, where in comparison to unformulated TA, TA-CCLs showed higher cell viability. In addition, clinical safety was further confirmed by measuring the corneal thickness and intraocular pressure [146].

In an interesting study by Li et al., the coating mechanism of cyclosporine liposomes by low molecular weight Cs and the effects of different coating variables such as molecular weight, concentration, and pH on coating efficiency were investigated using a mathematical model [147]. The authors also investigated the low molecular weight CCLs for their in vitro drug release characteristics, toxicity, as well as in vivo drug absorption and ocular bioavailability upon topical administration. In comparison to uncoated cyclosporine liposomes, low molecular weight CCLs showed a delayed drug release profile as well as facilitated drug internalization without affecting cell viability. Furthermore, the in vivo studies demonstrated a significant increase in cyclosporine concentrations in cornea, conjunctiva, and sclera when delivered topically in CCLs in comparison to plain uncoated liposomes. This positive effect of low molecular weight Cs coating on enhancing ocular bioavailability was explained by the mucoadhesive properties of chitosan which enable it to attach and interact with the negative charge of the mucus film and epithelia leading to prolonged ocular drug retention and permeation [147].

#### 5.2.3. Chitosan Coated Cubosomes

The tendency of amphiphilic lipids to self-assemble in water into well defined, highly organized and thermodynamically stable structures such as (1) the lamellar phases (L_α_), (2) the hexagonal phases (H_II_), and the bicontinuous cubic phases (Q_II_) (collectively known as lyotropic liquid crystal ((LLC) systems) presents many opportunities for drug delivery.

For example, the colloidal dispersion of a huge lamellar phase in water leads to the formation of liposomes which have been extensively investigated for a wide range of applications [148,149,150].

Cubosomes represent the nanostructured systems that result upon the colloidal dispersion of bicontinuous cubic liquid crystalline structures in aqueous media using suitable surfactants [148]. Cubosomes particle sizes range from 100 to 300 nm and are either formed spontaneously by self-assembly of amphiphilic lipids such as glyceryl monostearate in excess aqueous media or using high pressure emulsification of glycerol monooleate and water using suitable surfactants such as poloxamer 407 as a steric stabilizer of the bulk cubic phase lipid that has the appearance of rigid gels [151,152].

In comparison to the bilayer lamellar structure of liposomes, cubosomes display many distinct features which make them promising candidates for ophthalmic delivery. For example, in addition to being biocompatible, cubosomes are reported to be mucoadhesive [151]. In addition, their higher structural similarity with biological membranes enhances their fusion with the lipid bilayers of any mucosal epithelia. Furthermore, they have higher physical stability due to the larger ratio of lipid bilayers used and the presence of strong electrical repulsive forces. They also have higher specific surface area and good flowability at low viscosity while allowing for the incorporation of considerable amounts of hydrophilic, hydrophobic, as well as amphiphilic drugs [151].

Cubosomes have been investigated for topical delivery to ocular tissues, and have been shown to enhance ocular delivery while being safe and non-irritant. For example, Han et al. reported the preparation of low-irritant flurbiprofen cubosomes that displayed an increase in Tmax, the area under the curve, and mean residence time reflecting higher ocular bioavailability in comparison to flurbiprofen solutions [152]. In another study investigating the ophthalmic delivery of ketorolac using cubosomes, the authors reported a 2-fold increase in corneal retention and significantly increased transcorneal permeability when compared to ketorolac solution [153]. Timolol maleate cubosomes have also been studied for glaucoma, and were found to enhance the retention and intraocular pressure, lowering efficacy when compared to the commercial timolol maleate eye drops [154]. The use of topical brimonidine tartarate loaded cubosomes has also been investigated for the management of glaucoma, and was found to have superior ocular bioavailability to that of the market product Alphagan^®^P while being a prolonged release, non-irritant and compliant alternative [155].

Recently, Said et al. reported the employment of mucoadhesion using CS to control and enhance the ocular delivery from cubosomes loaded with voriconazole. First, the authors optimized the cubosomal formulations using the lipid monoolein and the surfactant Pluronic F127 by face centred central composite design. Factorial design was then used to investigate the cubosomal composition that displayed the smallest particle size, the highest zeta potential, with maximal drug loading, % entrapment efficiency and prolonged voriconazole release profile. Next, the optimal voriconazole cubosomes (formulated at 15% monoolein and 1.2% Pluronic F127) were rendered mucoadhesive through coating with 0.5 *w*/*v* % CS solution. The chitosan-coated cubosomes (CCCs) were then characterized for their physicochemical properties, mucoadhesive characteristics, and in vivo pharmacokinetics and ocular irritation studies. Mucoadhesion evaluation of CCCs was carried out through the assessment of the changes in particle size and zeta potential values of the negatively charged mucin following incubation with the CCCs. The mucin particle size and zeta potential values displayed a significant increase following incubation with CCCs reflecting aggregate formation upon electrostatic interaction between mucin and CCCs. Furthermore, the in vivo studies showed significantly higher concentration of CCCs in the vitreous humour (3.2 ng/mL) vs. drug suspension (0.21 ng/mL) at (*p*-value *<* 0.0001) indicating the ability of CCCs to penetrate deeply through the corneal membrane until reaching the vitreous humour [156].

#### 5.2.4. Chitosan Coated Lipid Particles SLNPs and NLCs

Lipid nanoparticles includes three types of particles, solid lipid nanoparticles (SLNPs), nanostructured lipid carriers (NLCs), and hybrid lipid nanoparticles [11]. The role of mucoadhesion using chitosan surface coating on posterior segment delivery of SLNPs and NLCs has been investigated by Balguri et al. [157]. In their study, the authors prepared and characterized indomethacin loaded SLNPs as well as NLCs which were then coated by low molecular weight chitosan (mol.wt. < 200 kDa). Surface coating by chitosan was confirmed by zeta potential measurements and was performed by adding the desired chitosan concentration to the aqueous phase before preparing the SLNPs and NLCs. The effect of chitosan surface coating on ocular bioavailability was then evaluated in vivo using tissue distribution studies in Male New Zealand White albino Rabbits. The results of in vivo studies demonstrated a strong correlation between surface coating of SLNPs and NLCs by chitosan and achieving higher indomethacin levels in deeper eye tissues such as RPE-choroid. Even though the highest indomethacin levels were achieved with chitosan coated NLCs, this could be attributed to their higher indomethacin loading. The dose of indomethacin in chitosan coated SLNPs was 10 fold lower than in chitosan coated NLCs, yet it delivered only 3–4 fold less indomethacin concentration to deeper eye tissues. This positive impact of chitosan on increasing indomethacin levels in anterior and posterior tissues was attributed by the authors to the documented mucoadhesive properties of chitosan and their ability to form electrostatic interactions with the ocular mucosa negatively charged sialic acid residues, increasing precorneal residence time. They also attributed their findings to the tendency of chitosan to generate a reversible sharp reduction in the trans epithelial electrical resistance (TEER) and promotion of model macromolecules permeability [157].

The effect of mucoadhesion on posterior segment eye delivery of lipid nanoparticles was also investigated in another study by Selvaraj et al. [158]. In their study, the authors investigated the unutilized antiangiogenic activity of itraconazole for DR treatment using mucoadhesive chitosan coated NLCs. First, the solubility of itraconazole in different solid and liquid lipids using partition coefficient studies was screened. Then, the Box–Behnken statistical design was used to optimize the formulation of itraconazole-loaded NLCs using the selected lipids and surfactants by hot high pressure homogenization. The effect of three independent factors namely (1) the total lipid ratio (mg), (2) the percent surfactant concentration, and (3) the number of high-pressure homogenization cycles on the resultant particle size and %EE was investigated. Surface coating of NLCs was then performed by dropwise addition of NLCs into (0.5% *w*/*v*) 20% acetylated CS solution while stirring. The CS coated NLCs were then evaluated for their size, surface charge, entrapment efficiency (EE), release properties as well as antiangiogenic potential. Successful surface coating by CS was confirmed by TEM analysis and particle size measurements which revealed an increase in particle size from 70.55 nm in uncoated NLCs to 86.75 nm in coated NLCs. Increased surface charge from −17.2 mV in uncoated NLCs to +25.6 mV in CS coated NLCs revealed successful surface coating with CS. The addition of CS to the surface of NLCs prolonged the itraconazole release profile which was attributed by the authors to the adhesion properties of CS that formed a hydrophilic matrix layer around NLCs, controlling drug release. The antiangiogenic potential and VEGF targeting efficiency of itraconazole-loaded NLCs were then investigated using ex vivo as well as in vivo studies. In the ex vivo CAM Assay, Cs coated NLCs demonstrated a higher antiangiogenic effect in comparison to itraconazole control through the downregulation of VEGF165 leading to the inhibition of abnormal blood vessels formation and proliferation. A similar antineovascularization effect of CS coated NLCs was observed in in vivo VEGF165-induced model rat models. These results were explained by the superior mucoadhesive characteristics of CS and its ability to interact with negatively charged mucins. Furthermore, the CS induced increase in formulation viscosity leads to reduction in precorneal loss and potentiation of penetration behind the cornea concluding the targeting potential of CS coated NLCs [158].

#### 5.2.5. Chitosan Coated Nanomicelles

Micelles are nano-sized colloidal carriers (10–200 nm) formed through the self-aggregation of amphiphilic block or graph copolymer in aqueous solutions. The micelle consists of a hydrophobic core and a hydrophilic shell and it is influenced by factors such as the mass and composition of the copolymer backbone, the concentration of the polymer chains and the properties of drug encapsulation [159]. The hydrophilic nature of the mucin layer covering the corneal and conjunctival epithelium in the eye provide it a protective barrier against the diffusion of hydrophobic molecules. Thus, mucoadhesive polymers have recently gained importance and are extensively explored to increase the bioavailability of the drug in the immobilized mucin layer and enhance the retention time.

Chitosan oliosaccharide (CSO), an oligomer of chitosan with average molecular weight (MW) < 10,000 Da, has exhibited the ability to increase the drug retention on the ocular surfaces owing owing to electrostatic interaction between chitosan’s positively charged amines and the negatively charged sialic acid remains of mucins. Xiaoyue et al. focused on nanomicelles formulated in eye drops for topical drug delivery to the posterior segment of the eye [5]. In their study, Dexamethasone (DEX) was used as a model drug for treating macula edema. They designed chitosan oligosaccharide-valylvaline-steric acid (CVS) nanomicelles formulated in eye drops to improve bioavailability in the posterior segments. Additionally, they also developed around 31 nm sized micellar formulations of DEX using a blend of polymers including polyoxyethylene hydrogenated castor oil 40 and octoxynol-40 (HCO-40/OC-40). For CVS nanomicelles, the synthesis of CVS polymers composed of 2 steps, which were copolymerization of CSO-SA and valylvaline (VV) conjugation to the CSO-SA block copolymers by lyophilization. Then, DEX was loaded into the CVS polymer solution to form nanomicelles via probe-type ultrasonic and dialysis methods. HCO-40/OC-40/DEX were prepared by a thin-film hydration method using ethanol as the organic solvent. In vitro cytotoxicity studies in human corneal epithelial primary cells (HCEpiC) and human conjunctival epithelial primary cells (HConEpiC) systems revealed that the cell viability of DEX loaded into both nanomicelles reached 80% after 12 h, showing no significant cytotoxicity. This might be ascribed to DEX encapsulation in the hydrophobic micelle core and slow release, which reduced drug concentration and toxicity compared with DEX liquid preparation alone. In addition, an ex vivo fluorescence study of the active transport of CVS nanomicelles by peptide transporter-1 (PepT-1) indicated CVS nanomicelles entered the posterior segment mainly through the conjunctival route owing to the larger conjunctival-scleral surface area, which allows lateral diffusion of CVS nanomicelles to reach the posterior part effectively [160]. Besides, in vivo distribution evaluation of rabbits’ eyes suggested DEX from both nanomicelles could be detected for more than 3 h in rabbit tears. In vivo distribution evaluation of rabbits’ eyes showed the delivering efficiency of CSV nanomicelles was not inferior to that of HCO-40/OC-40 mixed nanomicelles. The approximately 100 nm sized CVS nanomicelles with zeta potential of 33 mV and encapsulation efficiency (EE) 92% illustrated in vivo permeation of DEX (200 ng/g) to the scleral-choroid-retina in rabbits within 2 h. Additionally, due to the incorporation of valylvaline and steric acid (VV-SA, ratio 5:4) to the CSO nanomicelles, in vitro studies in simulated tear fluid demonstrated 60% of DEX release for up to 6 h, whilst CSO nanomicelles had only 40% of drug release in the same period of time.

Thus, these findings indicated that the CVS nanomicelles modification presented sustained release, biocompatibility and penetration enhancing properties that could become promising candidates for ocular drug delivery to the posterior segment of the eye [5].

#### 5.2.6. Chitosan Coated Nanocomposites

Nanocomposites are composite materials in which the matrix material is reinforced by one or more separate nanomaterials in order to improve performance properties. The multifunctional properties of these hybrid nanocomposites were attributed to active targeting, bioadhesive capacity and penetration enhancement. Carboxymethyl chitosan (CMCS) is highly soluble in water and the presence of active groups such as hydroxyl, amino and carboxyl make it a promising carrier for targeted modification and special targeting delivery to PepT-1 [161]. Although there is a lot of interesting and innovative work being published on mucoadhesives using cationic chitosan, there is only a small number of reports on improved chitosan coated nanocomposites [162,163].

Cao and co-workers developed a combination of organic-inorganic hybrid nanocomposites based on inorganic materials of layered double hydroxide (LDH) and organic materials of functional carboxymethyl chitosan (CMCS) derivatives for drug delivery to the posterior segment of the eye by topical administration [164]. Dexamethasone sodium phosphate (DEXP), a highly water-soluble drug, has been developed as an implant to inject into the vitreous to manage retinal diseases. In their study, a special substrate of peptide transporter-1 (PepT-1) and glutathione (GSH) was modified on CMCS. Valylvaline (VV) and glycylsarcosine (GS) have been proved to be reliable target ligands of PepT-1 in prodrugs, and thiolated carboxymethyl chitosan modified by glutathione (GSH) can exhibit a strong mucoadhesive property and a permeation enhancing effect. CMCS-glutathione-glycylsarcosine (CMCG-GS) and CMCS-glutathione-valylvaline (CMCG-VV)-LDH hybrid nanocomposites were prepared and structurally confirmed. Approximately 150–200 nm sized nanocomposites with zeta potential +30 mV and drug loading of DEXP ranging between 9 and 12% were obtained from nanocomposites hybridized with CMCG-GS and CMCG-VV. In vitro studies demonstrated a sustained drug release (35–65%) for up to 6 h from both CMCG-GS-DEXP-LDH and CMCG-VV-DEXP-LDH nanocomposites and the cumulative release amount of DEXP decreased as the increased amount of CMCS derivatives. For this reason, the release of CMCG-GS/VV-DEXP-LDH hybrid nanocomposites with a higher amount of CMCG-GS (10:1) was slower than that of CMCG-GS/VV-DEXP-LDH with ratio 1:5.

In addition, the in vitro studies on human conjunctival epithelial cells after 24 h showed that CMCG-GS/VV-DEXP-LDH (1:5) or CMCG-GS/VV-DEXP-LDH (10:1) nanocomposites did not exhibit significant cell cytotoxicity (LDH concentration ≤ 100.0 μg/mL) and eye irritation in rabbit tears for up to 6 h. In vitro human conjunctival epithelial cells (HConEpiC) permeability studies displayed an almost 2.75- fold increase in DEXP permeability of CMCG-GS/VV-DEXP-LDH (10:1) nanocomposites compared to CMCG-GS/VV-DEXP-LDH (1:5) formulation groups (P_app_ = 5.40 ± 1.24 cm/s × 10^−6^). Additionally, an in vivo precorneal retention study showed an 8.35-fold, 2.87-fold and 2.58-fold increase in AUC0–6 h, Cmax and MRT for CMCG-GS-DEXP-LDH (10:1) hybrid nanocomposite eye drops, respectively, compared to that of the commercial product. This supported the mechanism of adsorption-mediated endocytosis and PepT-1 mediated actively targeting transport. Besides, visualization of transport routes based on ex vivo fluorescein isothiocyanate isome (FITC)-loaded LDH hybrid nanocomposites studies confirmed that FITC could diffuse into the choroid-retina with the shelter of LDH and CMCG-GS. The presence of a strong fluorescence signal of FITC-conjugated LDH hybrid nanocomposites in the sclera revealed that integral LDH nanocarriers reached the sclera. Thus, the released DEXP molecule was predicted to pass through the Bruch’s membrane and diffuse into the retina. In addition, in vivo tissue distribution studies in rabbit’s eyes showed the retention of DEXP from CMCG-GS-DEXP-LDH (10:1) in the target of the choroid-retina for 3 h with final concentration up to 121 ng/g tissues, whereas DEXP concentration from commercial eye drops could sustain in the choroid-retina for only 30 min (200 ng/g tissues). Therefore, the multifunctional carboxymethyl chitosan derivatives-layered double hydroxide hybrid nanocomposites developed by this study remain a possibility for the efficient drug delivery to the posterior segment of the eye via non-invasive topical instillation.

**Table 3:** Approaches to enhance posterior eye delivery using chitosan-based drug delivery.

Drug Delivery System	Loaded Drug	Ref.
Chitosan-hyalouronic acid nanoparticles	Erythropoeitin	[131]
Emulsion	Coumarin-6	[135]
Microemulsion	TA/coumarin-6 for PK studies	[137]
Liposomes	TA	[105]
Liposomes	TA	[145]
Liposomes	Coumarin-6	[146]
Cubosomes	Voriconazole	[156]
SLNPs/NLCs	Indomethacin	[157]
NLCs	Itraconazole	[158]
Nanomicelles	Dexamethasone	[5,160]
Nanocomposites	DEXP	[161,164]

## 6. Other Mucoadhesives

### 6.1. Mucoadhesion Using Poly Vinyl Alcohol

Furthermore, in order to achieve good corneal contact time of a topical drug delivery system, viscosity enhancing polymers such as polyvinyl alcohol (PVA) are added to topical formulations to reduce precorneal drug clearance and improve the corneal contact time [165,166]. Fujisawa et al. designed surface modification diclofenac (DIC) loaded liposomes to improve their stability and achieve sustained drug delivery for targeting the retina [165]. In their study, the surface modification of liposome was achieved using polyvinyl alcohol (PVA) and its derivative bearing a hydrophobic anchor end of the molecule (PVA-R). Based on this strategy, it was observed that the PVA or PVA-R liposomes had higher physical stability and showed less particle aggregation. Owing to higher chain flexibility and good dispersion properties, PVA or PVR-coated liposomes demonstrated higher mucoadhesion, leading to inhibition of liposome aggregation before transporting the drug to the retina. Additionally, the drug delivery of DIC to the retina in rabbit eyes was significantly higher with both the PVA- and PVR-coated liposomes compared to the non-liposomal formulation. After eye drop administration, approximately 15 and 10 ng/g of the drug from the PVA and PVA-R liposomes, respectively, were detected in posterior retina-choroid, whereas less than 8 ng/g of DIC was detected in this layer from DIC ophthalmic solution. Hence, modification of the liposome with PVA or PVA-R enhanced the physical stability of DIC-loaded liposomes and PVA-R liposome displayed effective retinal delivery of DIC by promoting non-corneal drug penetration after eye drop administration.

### 6.2. Hyalouronic Acid Based Mucoadhesion

Over the years, an enormous amount of research has been carried out on ocular drug delivery systems for the treatment of ocular diseases, including cyclodextrins (CDs), a cyclic oligosaccharides with lipophilic inner cavities and hydrophilic outer surfaces. For this reason, in drug delivery system, CDs is a good option as complexing agents to enhance the aqueous solubility of hydrophobic active ingredients. Moreover, hyaluronic acid (HA), a linear polysaccharide that is found in the eyes, especially in the vitreous humour, and other human tissues, presents an excellent advantage in retaining moisture, which leads to keep the eyes lubricated, resulting in prevention of eye dryness [167]. Thus, CDs based inclusion complexation with macroparticles by addition of mucoadhesive polymer is an interesting strategy to enhance drug delivery through membrane barriers for a longer time.

Jansook et al. developed γ-cyclodextrin (γ-CD) and randomly methylated β-cyclodextrin (β-CD) to enhance solubility of celecoxib (CCB), which is a non-steroidal anti-inflammatory administered for AMD and DR [167]. By combination of these nanoaggregates with mucoadhesive polymers, such as hydroxypropyl methylcellulose (HPMC) or hyaluronic acid (HA), they obtained eye-drop formulations that demonstrated improvements in drug permeation through transcorneal and transscleral routes with no cytotoxicity shown. In vitro permeation studies on the mucin coated membrane revealed that an eye drop suspension containing γ-CD and 0.5% *w*/*v* HA had 28-fold higher CCB concentration in the mucin than that of suspension without HA (CCB concentration remained = 0.5%). In addition, ex vivo permeation studies in rabbits illustrated an increase in drug permeation through scleral tissues from the formulation containing randomly methylated β-CD and HA (Apparent permeation coefficient P_app_ = 0.57 ± 0.1 × 10^−2^ cm/h) in comparison to the formulation without HA, which the drug could not reach the scleral tissue of the rabbits. Thus, this can be attributed to the synergistic enhancement of transcorneal permeation caused by HA.

In a similar approach, Lorenzo-Veiga et al. prepared ocular nepafenac nanocarriers [168]. In their study, they used a combination of polymers (methylcellulose, carboxy methyl cellulose, and sodium hyaluronate) and hydroxypropyl β-cyclodextrin (HP-β-CD)/γ-cyclodextrin (γ-CD) to analyze C-based formulations with several polymers to efficiently deliver nepafenac topically to the eye in the form of microparticles. Their results also demonstrated formulations with HA showed improved solubility, mucoadhesion, permeability and anti-inflammatory potential as compared to a commercially nepafenac suspension. Thus, these provide an alternative for the topical delivery of hydrophobic drugs used to treat ocular diseases at the back of the eye by utilizing CDs and HA.

### 6.3. Alginate Based Mucoadhesion

Alginates are a group of naturally occurring nontoxic mucoadhesive gelling agents derived from a variety of brown seaweeds [169]. Gelation occurs when the alginate interacts with divalent cations of polyelectrolytes or proteoglycans, and the cations then bind to the anionic polysaccharide of the sodium alginate. In terms of its property, sodium alginate has been used as a viscosity enhancer for ophthalmic formulations. Babu and co-workers used the complex between nepafenac (NF), a water-insoluble nonsteroidal anti-inflammatory drug, and HP- β-CD to formulate an ion-activated in situ gel system using sodium alginate and protanal PH 1033 in order to enhance the residence time and reduce repeat eye drop instillation [170]. The result displayed that the viscosity of the formulation containing sodium alginate 0.5% *w*/*v* was increased 30-fold when exposed to the simulated tear fluid at 35 °C. Moreover, ex vivo transcorneal permeation studies of porcine eyes conferred greater permeation for drug loaded in situ gels containing 0.1–0.5% *w*/*v* sodium alginate in comparison to commercial NF, with at least 14-fold higher permeability rate compared to commercial NF eye drops in the porcine corneas.

Permeation studies indicated the drug concentration of the in situ formulations were around 10 times higher than the commercial product of NF. Besides, ex vivo ocular drug distribution studies in the porcine eyes further confirmed that the in situ formulations with sodium alginate not only acted as a solubilizing enhancer but also provided higher drug bioavailability in comparison to the aqueous drug suspension. The in situ gel formulation had a 5-fold increase in the NF concentration retained in the cornea compared to commercial NF. Finally, ex vivo drug distribution studies in isolated porcine sclera at 2 h revealed a higher drug permeability with in situ gels (10 µg/g of tissue) in comparison to a NF suspension (2 µg/g of tissue). Therefore, these studies indicated the potential of in situ gels in combination with alginate in improving the bioavailability of NF in the sclera following topical administration.

## 7. Challenges of Preclinical to Clinical Translation

Due to the numerous ocular barriers encountered, only upto 1/100,000th of topically applied drugs can be detected in retina and other posterior eye tissues. For this reason, it has always been perceived that topical drugs cannot access retinal tissues [15]. However, with the demonstration of topically applied drugs reaching the posterior eye segment through the corneal and the conjunctival pathways, this applicability is currently being reassessed [99,100,101,102,103,104,105]. As mentioned previously, the higher the residence time of topically applied drugs at the ocular surface, the greater the chances for drug permeation. Consequently, mucoadhesion is expected to enhance topical drugs absorption to the posterior eye segment as illustrated in Figure 6A.

The successful design of a mucoadhesive drug delivery system needs careful consideration of numerous factors. One of the most important things to consider is the drug’s physicochemical properties as well as therapeutic dose. It has been estimated that ideal drug candidates for mucosal delivery should exhibit a reasonable aqueous solubility (>1 mg/mL), log *p* value (1–2), and should have a molecular weight of less than 400–500 D [171]. Furthermore, the drug’s therapeutic daily dose cannot practically exceed 10 mg. This becomes particularly important in ophthalmic mucosal delivery, due to the small ocular surface area and the small tear volume (7 μL). This imposes restrictions on the formulation volume or surface area (in case of solid dosage forms) that can be administered. Thus, restricting drug-loading capability and limiting applicability to low dose potent drugs [109,171].

Another critical factor to consider is the strength of mucoadhesiveness of the prepared drug DDS. The design of a highly mucoadhesive system that can resist rapid washout is highly desirable. Polymer molecular weight, concentration, chemical structure, surface charge, surface tension, and rate of hydration are amongst the important factors that determine the polymer’s suitability for mucoadhesion. With respect to ocular mucoadhesion, extra care should also be taken to demonstrate safety, non- irritancy, lack of interference with visual activity, and high drug loading capacity. Furthermore, care should be taken to ensure complete drug release and absorption before ocular mucin’s turnover, which occurs every 15–20 h [109].

The importance of demonstrating sufficient mucoadhesion is crucial for the clinical translation of mucoadhesive DDSs especially in the case of ophthalmic delivery. This is not only because of the over exposure of the ocular route to several precorneal removal stresses, but also because of the limited thickness of its mucosal layer (0.05–1.5 µm), as well as the lack of blood supply to ocular mucus. All these factors necessitate the selection of highly mucoadhesive polymers that are capable of creating strong adhesive joints upon topical application. However, despite the presence of several mucoadhesion characterization techniques (as illustrated in Section 4.3), only a few studies have evaluated the mucoadhesive performance of their investigated formulations. In addition to demonstrating sufficient mucoadhesion, one of the most crucial determinants of the success of mucoadhesion in achieving posterior segment eye delivery is the presence of therapeutic drug concentrations in the retina following topical administration. However, most reviewed literature report in vitro and in vivo qualitative studies that do not give enough insight into the usefulness of mucoadhesion for posterior eye delivery. Thus, there is a crucial need for more well designed pharmacokinetic as well as pharmacodynamic studies to be conducted both in vivo and on the clinical level to take into account the structural complexity of the human eye. In addition to the above considerations, another important factor to consider for successful mucosal delivery is the effect of the interpersonal variability in ocular mucus thickness and secretions on drug’s absorption. Moreover, it is important to be able to predict the effect of pathological factors (as in dry eye syndrome) on mucins structure, production, and hence the mucoadhesive performance of the designed formula, its performance, and drug absorption [171]. Most of the reviewed literature that investigated mucoadhesion for posterior eye delivery used CS for its numerous merits including availability, abundance, cationic properties, safety, biocompatibility, biodegradability, and non-immunogenicity [19]. To exploit chitosan’s mucoadhesion potential in promoting posterior eye delivery, many factors need to be carefully considered and critically reviewed.

As previously discussed in Section 5, an acidic environment that maintains chitosan in its soluble and positively charged form is critical for keeping its mucoadhesive and permeation enhancement activities [17]. Thus, it is important to note that at the neutral pH of the ocular surface to which the chitosan-based drug delivery system will be applied, chitosan’s mucoadhesive and permeation enhancement activities are expected to be greatly compromised. In some similar situations, a potential solution would be to render the formulation slightly acidic. However, due to the hypersensitivity of the ocular tissues, these formulations will increase reflex blinking and lachrymation, which will in turn lead to increased precorneal clearance and dosage form removal from the ocular surface. Furthermore, the human tear buffering capacity can compromise this premise.

Another important factor to consider regarding the usage of CS for posterior eye delivery, is the extent to which chitosan can promote topical drug absorption to the back of the eye. Many literature reports use permeation studies across cell cultures to demonstrate permeability of their investigated formulae. However, unlike most other tissues, the eye is highly protected by static and dynamic barriers whose effects cannot be demonstrated in cell culture studies. Even in reports carrying out in vivo evaluation of their CS based systems, the majority of papers conduct qualitative in vivo studies using fluorescence studies and do not measure drug concentrations in the posterior segment tissues (vitreous/retina). The only study that did this found that the estimated cumulative drug amount reaching the vitreous after 3 weeks is estimated to be around 0.01% of the initially applied dose [105]. To evaluate the usefulness of these results, well designed pharmacodynamic studies are needed to determine if this ratio is sufficient in relieving the disease symptoms and if the used dosing interval is sufficient for chronic disease management.

For clinical translation and regulatory approval, in addition to having controlled in vivo PK and PD studies, it is also essential to demonstrate productive and reproducible drug absorption following topical administration, as well as validate the absorption pathway involved.

Taking into consideration that most of the studies in which the corneal and conjunctival absorption pathways have been demonstrated were obtained from animal studies in rodents, it is important to highlight the need for further pharmacological mechanistic studies in more clinically relevant models that show less ocular species variations with humans. These studies should be able to differentiate corneal from conjunctival absorption pathways, and quantify drug absorption through each while accounting for the higher structural complexity of the human eye. This is not only important to give insight into human ocular pharmacokinetics following topical delivery, but will also help to validate reproducible and productive absorption patterns required for regulatory approval.

Clinical translation of chitosan-based systems for posterior eye delivery also requires the validation of its exact mechanism of action. As discussed in this review, chitosan is not only a mucoadhesive polymer, but is also a permeation enhancer. Additionally, in many studies, chitosan was investigated along with other permeation enhancers such as B-cyclodextrin, and thus the results in these studies should be interpreted with caution. Therefore, it is important to have well designed experiments that can explain the extent by which each mucoadhesive and mucopenetration enhancer contribute to posterior eye delivery.

It is also important to note that although there is direct evidence of the involvement of tight junctions opening in the permeation enhancement mechanism of chitosan, most of this evidence has been obtained from both Caco-2 cells and in vivo mice studies [17]. A study investigating the detailed mechanism of chitosan’s permeation enhancement found that integrin αvβ3 that electrostatically interacts with chitosan to mediate its tissue permeation effect, is very highly expressed in the Caco-2 colorectal cell line in comparison to normal tissues. Thus, the permeation enhancement activity of chitosan should be considered with some caution. Moreover, the definite mechanism of chitosan’s permeation enhancement is yet to be fully elucidated [17]. For example, while most studies report that chitosan’s permeation involves the paracellular route, other studies suggest the involvement of endocytosis and increased cell metabolism. An excellent overview of these studies and their conflicting results is very well described in the literature [17].

Thus, for the utilization of chitosan’s permeation potential in ocular delivery, ocular specific mechanistic studies that determine extent and degree of chitosan’s permeation enhancement would be needed by regulatory authorities.

Another very important consideration for the clinical translation of CS-based systems is the demonstration of its safety and biodegradability. CS being a cationic polymer, it is expected to be toxic to the corneal as well as the conjunctival tissues. For this reason, numerous in vitro as well as in vivo studies investigated the toxicological profile of chitosan-based systems for ocular delivery [172,173,174,175,176]. It is important to denote that the safety profile of CS based systems is concentration as well composition dependant. For instance, many studies reported nearly 100% cell viability and survival for CS based systems with no signs of in vivo inflammation or alteration [173,174,176,177]. However, this low toxicity profile was corresponding to low CS concentrations not exceeding 2 mg/mL. Another important aspect is the effect of particle composition on toxicity. For example, chitosan liposome complexes showed higher cell viability than chitosan nanoparticles after cell incubation for 30 min [175]. Therefore, for every CS based DDS, it is required to demonstrate ocular safety and investigate the encountered degradation pathways, and if the degradation by-products are safe to the ocular tissues. In PSEDs, chronic administration of high doses of chitosan nanoparticles is expected to be required for long-term delivery of therapeutic drug concentrations to the posterior eye segment. Thus, one of the most critical issues to address is the short as well as long term safety at relevant CS doses.

Finally, an important challenge for the clinical translation of CS-based systems is to be able to demonstrate reproducible production of the polymer. Chitosan is not a single unique substance, but rather too many copolymers with a different ratio of residues [178]. Thus, having a defined and quality controlled chitosan grade is very important, as variability in chitosan physicochemical properties including molecular weight, degree of deacetylation, etc., does not only affect activity, but also mucoadhesion, permeation, and even biodegradability as well as in vivo performance.

## 8. Conclusions

Vision impairment is a global public priority that highly influences both developed as well as developing communities. Due to the increase in aging populations, the prevalence of posterior segment eye diseases including AMD and DR is increasing. These diseases are amongst the major causes of irreversible blindness that affect millions of people worldwide.

Despite the successful development of several promising therapeutic options for ocular diseases, the delivery of these treatments especially to the posterior eye tissues remains highly challenging. Owing to the presence of numerous barriers that hinder posterior eye drug delivery, IVT injections represent the primary route to deliver drugs to the posterior eye tissues. However, IVT injections are highly invasive and can cause serious complications including infection, cataracts, retinal detachment and vitreous haemorrhage. Among the investigated non-invasive delivery approaches, mucoadhesion is receiving increased attention due to its ability to both reduce precorneal clearance and increase precorneal residence time, i.e., increasing the drug concentration gradient across the ocular tissues, thus, creating higher chances for corneal and conjunctival drugs absorption to the posterior eye tissues following topical application.

In this review, the potential of mucoadhesion in promoting topical delivery to the posterior eye segment is assessed. First, a brief account of the eye’s anatomy and the main challenges that face topical drug absorption to the posterior eye is given. After this, the explored pathways that can be harnessed for topical drug absorption into the posterior eye segment are presented. Then, an account of the most common PSEDs including AMD and DR and their treatment options is given. For thorough understanding of mucoadhesion and assessment of its potential in promoting posterior eye delivery, an account of mucoadhesion, its theory, factors affecting mucoadhesion, considerations for ocular mucoadhesion and a wide range of mucoadhesion characterization techniques are presented.

Several mucoadhesive polymers including chitosan, hyalouronic acid, polyvinyl alcohol, and alginates have been investigated to promote topical drug delivery to the posterior eye segment. Among these polymers, CS received maximum attention due to its numerous merits and outstanding mucoadhesive and mucopenetrating properties. Several CS based systems including CS nanoparticles as well as a wide range of CS coated DDSs have been investigated to promote posterior eye delivery following topical administration. In this review, an overview of these studies is presented and the challenges of preclinical to clinical translation of CS based DDS for delivery to the posterior eye segment are discussed.

## Figures and Tables

**Table 1:** Properties of intravitreal anti-VEGF agents intended for the management of PSEDs.

Drugs	Molecular Weight	IVT Injection Dosage Regimen	Mechanism of Action	Reported Side Effects
Bevacizumab (Avastin^®^)	149 kDa	1.25 mg q 4 weeks [37]	Humanized anti-VEGF-A antibody binding all isoforms and biologically active degradation products [38]	Blurred vision, vitreous floaters and swelling of the cornea [37]
Ranibizumab (Lucentis^®^)	48 kDa	0.3 or 0.5 mg q 4 weeks [39]	Humanized anti-VEGF-A antibody binding fragment targeting all isoforms and biologically active degradation products [40]	Endopthalmitis, vitreous floaters and eye pain [39]
Aflibercept (Eylea^®^)	96.6 kDa	2 mg at weeks 0, 4 and 8 then q 8 weeks [41]	Chimeric protein binding all isomers of the VEGF-A family, VEGF-B and PGF [42]	Conjunctival haemorrhage, eye pain, vitreous detachment and floaters and ocular hypertension [41,43]
Brolucizumab (Beovu^®^)	26 kDa	6 mg q 12 weeks [44]	Single-chain antibody fragment inhibitor of VEGF-A isoforms [44,45]	Blurred vision, cataract, conjunctival hemorrhage, vitreous floaters and eye pain [44,46,47]
Conbercept (Lumitin^®^)	142 kDa	0.5 mg at weeks 1, 4, 12 and 24 [48]	Recombinant fusion protein targeting multiple VEGF isoforms (VEGF-B, PIGF and VEGF-A) [49]	Eye pain, intraocular pressure and conjunctival haemorrhage [49,50]

VEGF—Vascular Endothelial Growth Factors, PGF—Placental Growth Factors, IVT injection—Intravitreal injection.

**Table 2:** Theory governing the interactions occurring at the mucus/mucoadhesive polymers interface.

Theory	Mechanism	Illustration
Wettability theory	Used to describe the mucoadhesive interactions in case of liquid formulations based on their spreadability on mucosal membranes. Interfacial tension is the main determinant of wettability and spreadability of liquid formulations. In addition, other secondary factors including viscosity, disjoining pressure, and contact line friction effects are also reported to affect the wetting of surfaces [116]. The better the spreadability, the higher the chances for mucoadhesion. Spreadability can be assessed through the measurement of the liquid formulations’ contact angles with the mucosal surface using the equation (γ SG = γ SL + γ LG cos (θ C), where γ SG is the surface tensions of the solid, γ LG is the surface tension of the liquid, γ SL represents the interfacial tension between them, and θ is the contact angle formed when they come into contact. The lower the value of the contact angle, the higher the spreadability and vice versa, and hence the higher the chances of mucosal interactions [110].	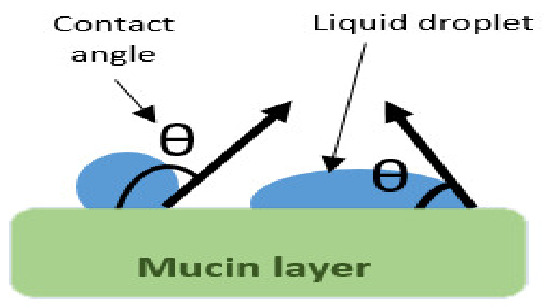
Mechanical theory	Used to describe mucoadhesion of liquids, it attributes mucoadhesion to the interlocking of the liquid mucoadhseive into the rough mucosal surface irregularities. Surface roughness increases the interaction area as well as viscoelastic and plastic dissipation of energy in case of joint failure. These later factors are considered more critical for mucoadhesion than the mechanical effect alone [107]	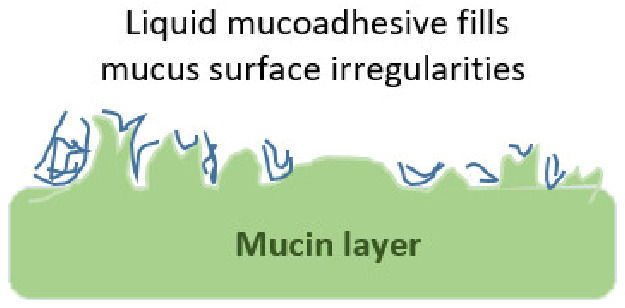
Electronic theory	Mucoadhesive interactions are attributed to the formation of electrostatic interactions between oppositely charged surfaces, as in the electrostatic interaction between the negatively charged mucin and positively charged polymers such as chitosan [110]	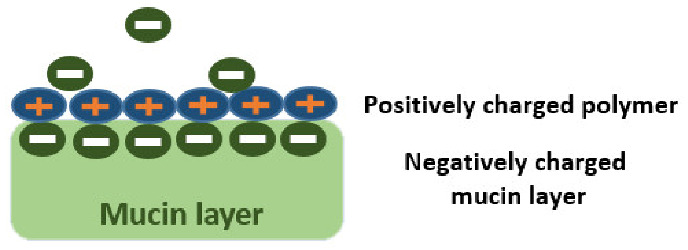
Adsorption theory	Mucoadhesion is a result of covalent bonding between the formulation and the mucus surface via hydrogen bonds and van der waal’s forces [110]	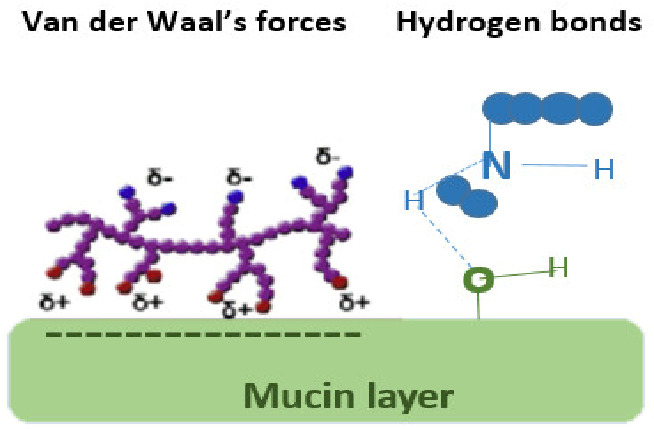
Diffusion interlocking theory	In this theory, mucoadhesion is attributed to time and concentration gradient dependent inter diffusion of polymer chains and mucus glycoprotein chains across the mucoadhesive interface. Following diffusion, sufficient interpenetration depth and chain entanglements induce semi-permanent mucoadhesive bond formation. This process is governed by several factors including temperature, polymer molecular weight, molecular chain length, cross-linking density, molecular chain mobility and flexibility, as well as expansion capacity [107,110]	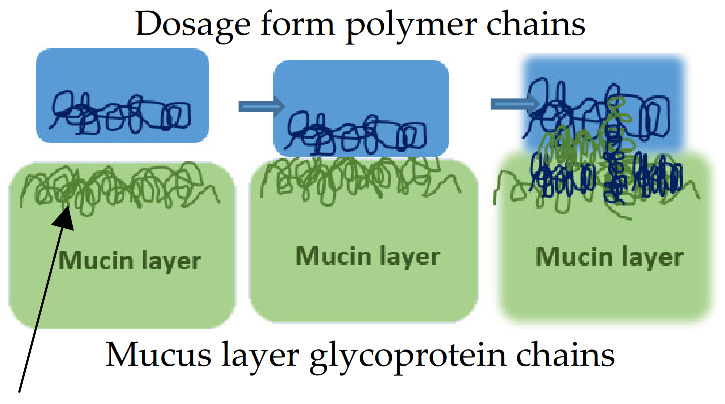
Fracture theory	It differs from the other theories in relating the strength of mucoadhesion between two surfaces to the force required for their separation and detachment. It is assumed that the mucoadhesive bond failure occurs at the interface; however, failure typically occurs at the weakest point which could be any of the adhering surfaces as well [107,110]	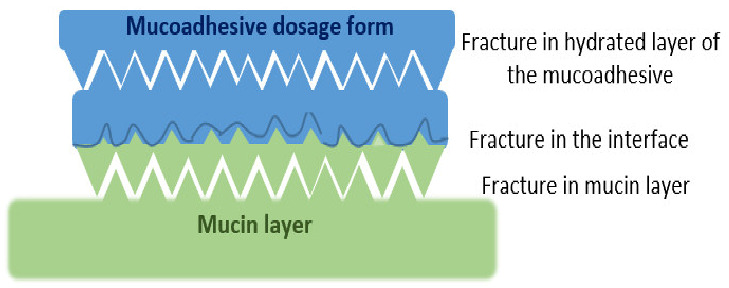

## Data Availability

Not applicable.

## Abbreviations

AFM	Atomic force microscopy
AMD	Age Macular Degeneration
Anti-VEGFs	Anti-vascular endothelial growth factors agents
ATR–FTIR	Fourier transform infrared spectral analysis
β-CD	β-cyclodextrin
CCB	Celecoxib
CCCs	Chitosan coated cubosomes
CCLs	chitosan coated liposomes
CDs	Cyclodextrins
CMCS	Carboxymethyl chitosan
CNV	Choroidal neovascularization (CNV)
Cs	Chitosan
CSO	Chitosan oligosaccharide
CVS	Chitosan oligosaccharide-valylvaline-steric acid
DEX	Dexamethasone
DR	Diabetic Retinopathy
DDS	Drug Delivery System
%EE	% Entrapment Efficiency
FITC	Fluorescein isothiocyanate isomer
GIT	Gastrointestinal tract
HConEpiC	Human conjunctival epithelial primary cells
HCEpiC	Human Corneal Epithelial Primary Cells
HP-β-CD	Hydroxypropyl β-cyclodextrin
HPMC	Hydroxypropyl methylcellulose
IVT	Intravitreal
LDH	Inorganic materials of hydroxide
MEs	microemulsions
MLVs	multilamellar vesicles
NF	Nepafenac
NLCs	Nanostructured lipid carriers
NMR	Nuclear magnetic resonance
OC-40	Octoxynol-40
OcDD	Ocular drug delivery
PDT	Photodynamic therapy
PEG	Poly (ethylene glycol)
PepT-1	Peptide transporter-1
PGF	Placental Growth Factors
P-gp	P-glycoprotein
PLGA	poly (lactic-*co*-glycolic acid)
PDS	port delivery system
PS	Particle size
PSEDs	Posterior segment eye diseases
PVA	Polyvinyl alcohol
PVA-R	Polyvinyl alcohol derivatives bearing a hydrophobic anchor at the terminate molecule
SA	Steric acid
TA	Triamcinolone aetonide
TER	Transepithelial electrical resistance
TPP	Sodium tripolyphosphate
ULVs	Unilamellar vesicles
VEGF	Vascular Endothelial Growth Factors
VV	Valyl valine
ZP	Zeta potential
